# Enhanced Solar Photothermal Catalysis over Solution Plasma Activated TiO_2_

**DOI:** 10.1002/advs.202000204

**Published:** 2020-06-11

**Authors:** Fei Yu, Changhua Wang, Yingying Li, He Ma, Rui Wang, Yichun Liu, Norihiro Suzuki, Chiaki Terashima, Bunsho Ohtani, Tsuyoshi Ochiai, Akira Fujishima, Xintong Zhang

**Affiliations:** ^1^ Key Laboratory of UV‐Emitting Materials and Technology of Chinese Ministry of Education Northeast Normal University Changchun 130024 China; ^2^ Photocatalysis International Research Center Research Institute for Science & Technology Tokyo University of Science 2641 Yamazaki Noda Chiba 278‐8510 Japan; ^3^ Graduate School of Environmental Science Hokkaido University Sapporo 060‐0810 Japan; ^4^ Materials Analysis Group Kawasaki Technical Support Department Local Independent Administrative Agency Kanagawa Institute of industrial Science and Technology (KISTEC) Kanagawa 213‐0012 Japan

**Keywords:** oxygen vacancies, photothermal catalysis, solar energy, solution plasma

## Abstract

Colored wide‐bandgap semiconductor oxides with abundant mid‐gap states have long been regarded as promising visible light responsive photocatalysts. However, their catalytic activities are hampered by charge recombination at deep level defects, which constitutes the critical challenge to practical applications of these oxide photocatalysts. To address the challenge, a strategy is proposed here that includes creating shallow‐level defects above the deep‐level defects and thermal activating the migration of trapped electrons out of the deep‐level defects via these shallow defects. A simple and scalable solution plasma processing (SPP) technique is developed to process the presynthesized yellow TiO_2_ with numerous oxygen vacancies (Ov), which incorporates hydrogen dopants into the TiO_2_ lattice and creates shallow‐level defects above deep level of Ov, meanwhile retaining the original visible absorption of the colored TiO_2_. At elevated temperature, the SPP‐treated TiO_2_ exhibits a 300 times higher conversion rate for CO_2_ reduction under solar light irradiation and a 7.5 times higher removal rate of acetaldehyde under UV light irradiation, suggesting the effectiveness of the proposed strategy to enhance the photoactivity of colored wide‐bandgap oxides for energy and environmental applications.

## Graphical abstract

Solution plasma brings H doping into oxygen vacancy type TiO_2_. The H dopant bridges the gap between the oxygen vacancy (Ov) and conduction band (CB), resulting in photothermal extraction of trapped electrons at the Ov to CB. The solution plasma strategy endows a 300 times higher conversion rate for CO_2_ reduction under solar light at 393 K than that at 298 K.

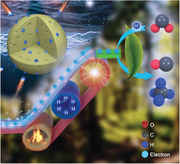

Solar‐driven photocatalytic processes attract substantial attention because they offer significant energy savings and because of their environmental friendliness.^[^ ^1^, ^2^, ^3^, ^4^, ^5^ ^]^ Recently developed solar‐induced photothermal catalysis demonstrated significantly better results than solar‐induced photocatalysis alone,^[^ ^6^, ^7^, ^8^, ^9^ ^]^ especially when performed using semiconductor oxide catalysts enriched with defects.^[^ ^10^, ^11^, ^12^, ^13^ ^]^ The defect at surface can facilitate the adsorption and activation of substrate molecules to boost the sluggish reaction thermodynamics and kinetics.^[^ ^14^, ^15^ ^]^ Defects may also activate lattice oxygen, which participates in the photothermal coupling catalysis.^[^ ^16^, ^17^, ^18^ ^]^ The electrons can be trapped at defects and thermally released to the surface, which, in turn, improves charge utilization and accelerates reaction rate at the surface. These advantages of defects are the major factors behind the recent renewed interest to semiconductor oxide photocatalysts in solar‐induced photothermal processes.

Defect engineering has been intensively investigated on TiO_2_ materials in past decades for pursuing visible light photocatalysis. Oxygen vacancies (Ov) are the most exploited defects of TiO_2_, which as the native defects of oxides, can be easily incorporated into the lattice of TiO_2_ by annealing in oxygen‐deficient atmosphere or as a co‐product of anion or cation doping.^[^ ^19^ ^]^ Enriched Ov can redshift the absorption spectra of TiO_2_ and changes its color from white.^[^ ^20^, ^21^, ^22^, ^23^, ^24^ ^]^ However, the Ov levels, ≈0.75 eV below the conduction band bottom of TiO_2_, are deep levels and work more likely as recombination centers,^[^ ^25^, ^26^ ^]^ which impairs the photocatalytic activity of colored TiO_2_. Hydrogen‐doping are another important means to modify TiO_2_ photocatalysts.^[^ ^27^, ^28^, ^29^ ^]^ Hydrogen is a shallow donor in TiO_2_, with defect levels closer to the conduction band of TiO_2_ than Ov.^[^ ^30^, ^31^, ^32^, ^33^ ^]^ Hydrogen‐doping is reported to enhance the photocatalytic activity of TiO_2_, and even produces black TiO_2_ under severe conditions by the formation of amorphous TiO_2_:H or defect complex.^[^ ^34^ ^]^ It is interesting to note that incorporating shallow hydrogen‐donor levels above Ov deep levels might be a promising way to enhance the photocatalysis over Ov‐TiO_2_, by which the trapped electrons can be extracted out of Ov deep levels via the shallow levels bridge, and the enhancement should be more remarkable for the photothermal catalysis due to the thermal activation of electron extraction. However, up to now few reports have investigated the synergistic effect of Ov and hydrogen dopants on enhancing the visible light photocatalysis of TiO_2_. And to the best knowledge of us, almost no report has dealt with the synergistic effect of Ov and hydrogen dopants on solar photothermal catalysis over TiO_2_.

Herein, we report that the solar photothermal catalysis over colored Ov‐rich TiO_2_ is indeed enhanced by incorporating shallow donor levels of hydrogen. We employ a novel solution plasma processing (SPP) to do hydrogen doping of Ov‐rich TiO_2_. Plasma‐in‐liquid can produce high flux of hydrogen radicals via H_2_O splitting at the open end of the discharge zone of SPP.^[^ ^35^, ^36^ ^]^ In comparison with conventional plasma‐in‐gas, SPP can be performed at room temperature and normal pressure,^[^ ^37^, ^38^, ^39^ ^]^ without the direct use of dihydrogen. The Ov‐rich TiO_2_ treated is a home‐made yellow‐colored TiO_2_, named as TiO_2_(AB), which is a mixture of anatase and TiO_2_(B) phases with anatase‐TiO_2_(B) heterojunction for efficient charge separation.^[^ ^40^ ^]^ As‐synthesized TiO_2_(AB) phase absorbed visible light up to 700 nm due to the presence of rich Ov. SPP treatment realized hydrogen doping into this Ov‐TiO_2_, which retained its visible light absorption and promoted charge separation. Activity of SPP‐treated TiO_2_(AB) in relationship to acetaldehyde degradation and CO_2_ reduction was significantly enhanced under both UV and visible light irradiation. Particularly, CO_2_ conversion rate can be enhanced by two orders of magnitude for photothermal catalytic reduction of CO_2_. We believe that SPP technique demonstrated in this work can be employed as a general strategy to activate other Ov‐rich wide‐bandgap semiconductor oxides, which are colored but weak in visible light photocatalysis, for solar photothermal catalysis applications.

Our goal was to incorporate as many oxygen vacancies into TiO_2_ as possible because TiO_2_ enriched with oxygen vacancies (Ov) is a prerequisite for achieving simultaneously strong visible light absorption. Thus, we synthesized TiO_2_ with oxygen vacancies by a solvothermal method and then treated the resulting product with SPP to incorporate hydrogen doping into this TiO_2_ (**Figure** **1**a). Ov locates at a deep energy level, the gap between Ov and conduction band of TiO_2_ is too wide to allow thermal excitation of trapped electron at Ov in photothermal catalysis. Thus, the role of the second step, SPP post‐treatment, is to introduce shallow energy level via hydrogen doping and fine‐tune the gap between Ov and conduction band of TiO_2_.

**Figure 1:**
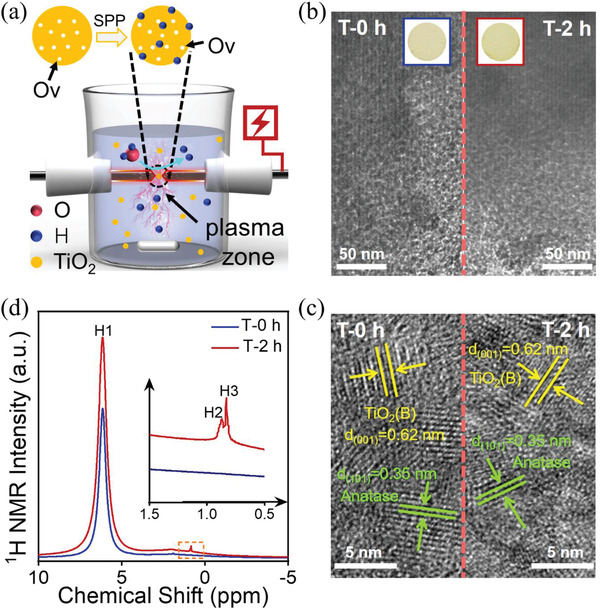
a) Schematic Illustration of the SPP Technique. b) TEM and c) HRTEM images of TiO_2_(AB) treated by SPP for 0 and 2 h (samples T‐0 h and T‐2 h, respectively). d) ^1^H NMR spectra of T‐0 h and T‐2 h, respectively.

As‐synthesized yellow TiO_2_ (Figure 1b, left, inset) demonstrated extended light absorption up to 700 nm (Figure 3a), which is a key to high catalytic activity under visible light. Transmission Electron Microscope (TEM) (Figure 1b, left) shows that as‐synthesized TiO_2_ had uniform particle sizes ≈10 nm in diameters. High Resolution Transmission Electron Microscope (HRTEM) (Figure 1c), X‐ray diffraction (XRD) and Raman (Figure S1, Supporting Information) analyses confirmed that the resulting sample consisted of anatase and TiO_2_(B) phases. This TiO_2_(AB) had high surface area (136 m^2^ g^−1^) as well as high porosity with the average pore size equal to 3.5 nm (Figure S2, Supporting Information). Electron Spin Resonance (ESR) spectrum showed a signal at *g* = 2.003 (Figure S3, Supporting Information), which is related to the interaction of surface Ov with adsorbed atmospheric O_2_.^[^ ^21^, ^25^ ^]^ This confirms the presence of Ov in the as‐synthesized yellow TiO_2_(AB). Thus, prior to SPP treatment, TiO_2_(AB) synthesized by solvothermal method contained abundant oxygen vacancies. Yang and coworkers also reported that creating Ov activated new catalytic sites and/or promoted the exposure of active sites. Accordingly, the catalytic performance with augmented reaction sites by the introduction of Ov vacancies will exceed that of the pristine catalyst.^[^ ^15^ ^]^

To understand structural changes occurring with TiO_2_(AB) after SPP treatment, it was characterized using combination of techniques. After SPP, TiO_2_(AB) became darker (Figure 1b, right, inset), however, TEM showed no obvious changes in dispersion and size of TiO_2_(AB) particles (Figure 1b, right). HRTEM (Figure 1c), XRD, Raman and BET (Figures S1 and S2, Supporting Information) analyses also showed no obvious changes in phase, structural and chemical composition of the sample as well as in its surface area. Therefore, SPP treatment is mild, nondestructive for the bulk structure, but probably modifies the surface structure. This is expected to enhance solar‐induced photocatalytic and photothermal catalytic activity of TiO_2_(AB), which will be discussed below (Vide infra).

Compared the ESR spectra of TiO_2_(AB) before and after SPP treatment (Figure S3, Supporting Information), the signal originated from Ov decreases after SPP treatment. This indicates the decrease of Ov content in TiO_2_(AB).^[^ ^41^ ^]^ As reported, plasma‐in‐liquid can instantaneously generate high temperature radiation (up to 10^4^–10^5^ K), which can eliminate some Ov.^[^ ^37^ ^]^ Herein, it is believed that the instant high temperature in liquid plasma contributes to the decrease of Ov content during the SPP treatment.

In high resolution of X‐ray photoelectron spectroscopy (XPS) spectra of Ti 2p (Figure S4a, Supporting Information), a slight shift higher binding energy from 458.75 to 458.80 eV has been detected. Compared the high resolution XPS spectra of O 2p before and after SPP treatment (Figure S4b, Supporting Information), the peak located at 530.14 eV can be assigned to Ti‐O for as‐synthesized yellow TiO_2_(AB). After SPP treatment, there is a subtle shift to 530.16 eV of Ti‐O. Additionally, the peak assigned to Ti‐OH synchronously shifts to higher binding energy. Moreover, the relative content of Ti‐OH is increased from 26.68% to 30.64%, indicating that H is effectively doped into the lattice.^[^ ^42^ ^]^ To further address this point, solid ^1^H NMR measurements were conducted (Figure 1d). Both samples show a large peak of H1 at chemical shift of 6.15 ppm which attributed to the characteristic bridging proton.^[^ ^27^, ^29^, ^43^ ^]^ The slightly larger line width in TiO_2_(AB) after SPP treatment may be caused by the incorporation of H atoms at bridging sites at the disordered phase produced during the SPP treatment process. Additionally, the stronger peak at 6.15 ppm suggests stronger hydrogen‐bonded bridging hydroxyl groups in TiO_2_(AB) after SPP treatment.^[^ ^28^ ^]^ In contrast to pristine TiO_2_(AB), TiO_2_(AB) after SPP treatment shows that two additional sharp resonances of H2 and H3 at chemical shifts of 0.82 ppm and 0.87 ppm are observed on the baseline. The additional signals can be assigned to the terminal and internal hydroxyl groups of TiO_2_, respectively, which are associated with hydrogen located in TiO_2_(AB) as a result of hydrogenation. In combination with above analysis, the SPP treatment not only gives rise to a slight decrease of Ov, but also realize hydrogen doping into TiO_2_(AB). The introduced H‐doped into TiO_2_(AB) lattice can make the color darker, which had same phenomenon in the previous studied about H‐doped TiO_2_.^[^ ^31^, ^43^ ^]^

To evaluate feasibility of SPP treatment as a strategy to enhance TiO_2_(AB) catalytic activity and to test applicability of the resulting TiO_2_(AB), we conducted two reactions: 1) degradation of gaseous acetaldehyde and 2) reduction of CO_2_ using irradiation by UV, visible, solar, blue, and green light. Photocatalytic tests were performed at room temperature. Photothermal catalytic tests were performed at different temperatures depending on the reaction enthalpy. Degradation of gaseous acetaldehyde is an exothermic reaction, thus, elevated temperatures are not favorable for the direct reaction to proceed. Therefore, photothermal catalytic reactions involving gaseous acetaldehyde were performed at 343 K. Because CO_2_ reduction is an endothermic reaction, its temperature during photothermal tests was maintained at 393 K.^[^ ^18^ ^]^

Photo‐ and photothermal catalytic reduction of CO_2_ was tested under solar and visible light irradiation. Reaction rates, at which CO and CH_4_ were generated over differently prepared catalysts, are shown in **Figure** **2**a,b and **Table** **1**. During the photocatalytic reaction performed using solar light, as‐prepared TiO_2_(AB) (named T‐0 h) demonstrated some activity judging by the amount of generated CO and CH_4_. These rates were 7.9 and 8.2 times higher, respectively, when CO_2_ reduction reaction was performed over TiO_2_(AB) treated by SPP for 2 h (named T‐2 h). Thus, T‐2 h enhanced its catalytic activity toward photoinduced reduction of CO_2_.

**Figure 2:**
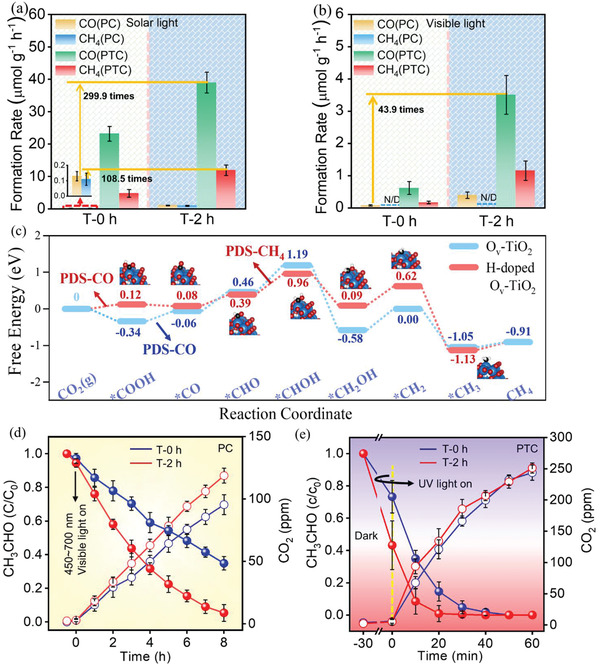
Photo‐ and photothermal catalytic activities of CO_2_ conversion over TiO_2_(AB) treated with SPP for 0 and 2 h (samples T‐0 h and T‐2 h, respectively) upon a) 100 mW cm^−2^ solar and b) visible (*λ* ≥ 420 nm) light irradiation (N/D: not detected); c) Free energy diagrams for CO_2_ reduction to CH_4_ by the thermochemical model on Ov‐TiO_2_ and H‐doped Ov‐TiO_2_. Insets are the corresponding structures of reaction intermediates for H‐doped Ov‐TiO_2_, where the blue, red, black, and white balls represent Ti, O, C, and H atoms, respectively. PDS: potential determining steps. Acetaldehyde degradation and CO_2_ evolution over T‐0 h and T‐2 h: d) under visible light at room temperature and e) under UV light with heating at 70 °C.

**Table 1:** Photo‐ and photothermal catalytic activities (PC and PTC, respectively) of CO_2_ conversion over TiO_2_(AB) treated by SPP for 0 and 2 h (samples T‐0 h and T‐2 h, respectively) upon a) 100 mW cm^−2^ solar and b) visible (*λ* ≥ 420 nm) light irradiation (N/D: not detected). Rate of reacted electrons (RRE) is equal to 2 r (CO) +8 r (CH_4_)

		PC			PTC			
Light	Catalyst	CO‐yield [µmol g^−1^ h^−1^]	CH_4_‐yield [µmol g^−1^ h^−1^]	RRE [µmol g^−1^ h^−1^]	CO‐yield [µmol g^−1^ h^−1^]	CH_4_‐yield [µmol g^−1^ h^−1^]	RRE [µmol g^−1^ h^−1^]	Selectivity of CH_4_ [%]
Solar light	T‐0 h	0.13	0.11	1.14	23.1	4.79	84.64	45.3
	T‐2 h	1.03	0.09	2.78	38.99	11.93	173.42	55.0
Visible light	T‐0 h	0.08	N/D	0.16	0.62	0.17	2.6	52.3
	T‐2 h	0.395	N/D	0.79	3.51	1.16	16.3	56.9

Both samples, T‐0 h and T‐2 h, demonstrated activity during photothermal catalytic reactions induced by solar light. Activities of T‐0 h and T‐2 h were on the different order of magnitude as their activities during corresponding photocatalytic reactions. Apparently, CO and CH_4_ formation rates over T‐2 h were 300 and 108 times higher during photothermal reactions, respectively, than those during photocatalytic reaction over T‐0 h. Compared with other TiO_2_, the yield of CO and CH_4_ are at a high level (Table S1, Supporting Information). Besides CO and CH_4_, other products such as C_2_H_4_, C_2_H_6_, C_3_H_6_, and C_3_H_8_ can be detected (Figure S5, Supporting Information). However, in comparison with CO and CH_4_, the yields of other products are very trace. CO_2_ reduction by H_2_O vapor mainly produces CO and CH_4_ and whereas CO_2_ reduction by H_2_O liquid mainly produces CH_3_OH. In our work, the photothermal catalytic reduction of CO_2_ is performed in H_2_O vapor. CO and CH_4_ are main products, which is in consistence with previously reported (Table S2, Supporting Information). More importantly, an *m*/*z* of 29 is definitely detected, which can be solely produced via reduction of ^13^CO_2_ (Figure S6, Supporting Information).

To further analyze CO_2_ reduction efficiency and the selectivity of products, rate of the reacted electrons (RREs) and the selectivity of CH_4_ were calculated taking into account formed products:^[^ ^44^ ^]^

(1) $$\begin{array}{c} \mathrm{Rate}\;\mathrm{of}\;\mathrm{reacted}\;\mathrm{electrons}=2r\left(\mathrm{CO}\right)+8r\left(CH_{4}\right) \end{array}$$

(2) $$\begin{array}{c} \mathrm{Selectivity}\;\mathrm{of}\;CH_{4}=8r\left(CH_{4}\right)/\left\{2r\left(\mathrm{CO}\right)+8r\left(CH_{4}\right)\right\} \end{array}$$

Rate of reacted electrons over T‐2 h during photothermal catalysis (equal to 173.42 µmol g^−1^ h^−1^) was 152 times higher than rate over T‐0 h photocatalyst (Table 1). Thus, SPP treatment of yellow defective TiO_2_(AB) is an efficient strategy capable to enhance both photo‐ and photothermal catalytic properties. SPP treatment enhanced photothermal catalytic properties of TiO_2_(AB) more than its photocatalytic properties. The yield of CO and CH_4_ is too low to accurately evaluate the selectivity in the photocatalytic test. Accordingly, the selectivity is not discussed in the case of photocatalysis. As for the photothermal catalytic test, the solution plasma treatment of TiO_2_ improves CH_4_ selectivity from 45.3% to 55.0% under solar light and 52.3% to 56.9% under visible light, respectively. In spite of the improved selectivity over solution plasma activated TiO_2_, the increased magnitude is slight and inferior to the increase in rate of reaction electrons. Herein, it is difficult to conclude that solution plasma treatment can simultaneously improve the reaction rate and product selectivity of CO_2_ reduction. It should be noted that this work studies pure TiO_2_ and turns to no cocatalyst. In our previous work,^[^ ^18^ ^]^ we also compared the selectivity of Ov‐TiO_2_ in photocatalytic and photothermal catalytic reduction of CO_2_. The results similarly revealed that the selectivity of CH_4_ can be hardly improved over pure TiO_2_. However, the grafting of cocatalyst (e.g., CoO*_x_*, CuO*_x_*) can significantly improve the CH_4_ selectivity in photothermal catalytic reduction of CO_2_. The selectivity of CH_4_ is expected to be significantly improved in the future work on cocatalyst loaded TiO_2_ rather than pure TiO_2_.

Photo‐ and photothermal catalytic reactions upon exposure to visible light were studied to understand contribution of visible light spectrum to the reaction outcome upon exposure to full solar irradiance spectrum. Activities of the catalysts upon their exposure to the visible light showed the same trend as upon exposure to the full solar spectrum (Figure 2b). Under visible light, CO was the main product during photocatalytic reduction of CO_2_. Formation rate of CO over T‐2 h increased up to 4.9 times in comparison to the catalyst reaction performed over T‐0 h. Photothermal catalytic reactions resulted in simultaneous formation of CO and CH_4_ upon exposure to the visible light. Formation rates of CO and CH_4_ over T‐2 h were 43.9 and 21.7 times higher, respectively, in comparison to the reactions performed using T‐0 h photocatalyst.

The stability of T‐2 h toward CO_2_ reduction is evaluated in ten cycles (Figure S7, Supporting Information). As can be seen, the yield of CO and CH_4_ is decreased before the 5th cycle. However, the yield is maintained at a stable level after the 5th cycle, suggesting the good stability for practical application. Moreover, the spent photocatalyst of T‐2 h was characterized by XRD, Raman, XPS and ESR (Figures S8–10, Supporting Information). No obvious structural change can be observed after cycling tests, again confirming the good stability of T‐2 h. The better stability of T‐2 h may benefit from hydrogen doping. As is reported, the hydrogen doped oxides can be of good stability due to the formation of stable Ov‐H. The Ov‐H binding is more stable than either interstitial hydrogen or oxygen vacancy.^[^ ^45^, ^46^ ^]^ This tends to avoid the filling of oxygen vacancy by atmospheric oxygen.

To investigate the reactivity fundamental of the H‐doped O_v_‐TiO_2_ in photocatalytic CO_2_ reduction, the theory calculations of reaction pathway were performed. We construct two stable structural models: (101) surfaces of anatase TiO_2_ with oxygen vacancies (Ov‐TiO_2_) and (101) surfaces of H‐doped anatase TiO_2_ with oxygen vacancies (H‐doped Ov‐TiO_2_), referring to previous studies (Figure S11–S13, Supporting Information).^[^ ^47^, ^48^ ^]^ Figure 2c shows the calculated diagrams of stepwise Gibbs free energy for CO_2_ reduction into CH_4_. With regard to the reaction pathway from CO_2_ to CO, the Ov‐TiO_2_ and H‐doped Ov‐TiO_2_ display different potential determining steps (PDS). That is, the PDS of Ov‐TiO_2_ is from *COOH to *CO with an energy barrier of 0.28 eV, while the PDS of H‐doped Ov‐TiO_2_ is from CO_2_ to *COOH with an energy barrier of 0.12 eV. By comparison, the lower energy barrier of PDS for CO product over H‐doped Ov‐TiO_2_ indicates a faster reaction rate than that of Ov‐TiO_2_. Moreover, the initial step over H‐doped Ov‐TiO_2_ is of positive energy barrier, suggesting the increased reaction rate at elevated temperature. On the contrary, the initial step over Ov‐TiO_2_ is of negative energy barrier, which is thermodynamically unfavorable for enhanced reaction rate at elevated temperature. On the basis of above lower energy barrier and endothermic reaction characteristics over H‐doped Ov‐TiO_2_, it is reasonable to understand the more significant promotion of CO production over H‐doped Ov‐TiO_2_ in photothermal catalysis. With regard to the reaction pathway from CO_2_ to CH_4_, hydrogenation of *CHO to *CHOH is of the highest energy barrier and becomes the potential determining steps over both Ov‐TiO_2_ and H‐doped Ov‐TiO_2_. By further comparison, H‐doped Ov‐TiO_2_ exhibits lower energy barrier (0.57 eV) than that of Ov‐TiO_2_ (0.73 eV). Therefore, the lower energy barrier of PDS for CH_4_ can better explain the enhanced CH_4_ yield over H‐doped Ov‐TiO_2_ than Ov‐TiO_2_ in photothermal catalytic experiment. In order to further unravel the reaction mechanism at the atomic level, we also calculate and discuss the transition state for *CHO by adding *H to *CHOH (PDS‐CH_4_) over both Ov‐TiO_2_ and H‐doped Ov‐TiO_2_ (Figure S14, Supporting Information). The activation energy (Ea) of H‐doped Ov‐TiO_2_ is 0.86 eV, which is much lower than that of Ov‐TiO_2_ (2.29 eV). This decreased energy barrier of H‐doped Ov‐TiO_2_ agrees with the result that H‐doped Ov‐TiO_2_ is more conducive to CO_2_ reduction reaction in comparison with Ov‐TiO_2_.

Acetaldehyde degradation and CO_2_ evolution over TiO_2_(AB) with (for 2 h) and without SPP treatment are performed. Apparent rate constants (*k*) of acetaldehyde degradation are listed in **Table** **2**. During photocatalytic reaction T‐2 h demonstrated faster acetaldehyde removal rate and CO_2_ evolution than T‐0 h (Figure 2c and Figure S15, Supporting Information). Thus, T‐2 h provided an enhanced acetaldehyde degradation, which was observed upon exposure to UV, visible, blue and green light. Compared with commercial P25, the acetaldehyde removal rate of T‐2 h is faster in both UV and visible light (Figure S16, Supporting Information). The ESR analysis is studied under UV and visible light irradiation, respectively. This signal can be enhanced under UV and visible light irradiation, respectively, indicating sample T‐2 h can be active under not only UV but also visible light irradiation (Figure S17, Supporting Information). As observed in the photoactivity test (Figure S15a and Figure 2c, Supporting Information), the sample T‐2 h is active under both UV and visible light irradiation. The enhanced ESR signal under UV and visible light irradiation agrees with the as‐observed catalytic activity of T‐2 h under UV and visible light irradiation, respectively.

**Table 2:** BET surface areas and kinetic constants of TiO_2_(AB) treated by SPP for 0 and 2 h (samples T‐0 h and T‐2 h, respectively) used for photocatalytic degradation of acetaldehyde under UV (with and without heating at 70 °C), visible, blue, and green light irradiation

	Surface area [m^2^ g^−1^]	*k* (UV) [min^−1^]	*k* (Visible) [h^−1^]	*k* (Blue) [h^−1^]	*k* (Green) [h^−1^]	*k* (UV‐70 °C) [min^−1^]
T‐0 h	137	0.0256	0.124	0.402	0.117	0.0807
T‐2 h	134	0.0996	0.352	1.298	0.291	0.1917

Apparent rate constant for the photothermal catalytic reaction with T‐2 h was 7.5 times higher than the constant for the photocatalytic reaction with T‐0 h as a catalyst (Table 2). The surface temperature of T‐0 h and T‐2 h were closed to the heating temperature. This indicates that the higher performance of T‐2 h is not contributed by the thermal effect from light source (Figure S18, Supporting Information).^[^ ^49^ ^]^ Acetaldehyde removal over T‐2 h in the dark was 60% during the first 30 min (Figure S19, Supporting Information), which indicates an enhanced oxidation capacity of lattice oxygen in TiO_2_ treated by SPP. Stability of SPP treated TiO_2_(AB) was also evaluated via UV light photocatalysis (Figure S20, Supporting Information). Photocatalytic activity of T‐0 h after five cycling tests decreased by 75% while T‐2 h maintained its initial photocatalytic activity. T‐2 h demonstrated no activity loss after exposure to air for 80 d and after additional six cycling tests, which demonstrates excellent stability of SPP‐treated TiO_2_(AB).

We also measured apparent quantum efficiency (AQE) during the photocatalytic acetaldehyde degradation to determine solar energy conversion efficiency at different single wavelengths. AQEs for acetaldehyde degradation at 365 nm were 4.1% and 12.8% when T‐0 h and T‐2 h were used, respectively (Figure S21, Supporting Information). AQE of acetaldehyde degradation in the visible light region with T‐2 h as a catalyst was ≈1%. Thus, based on all results mentioned above on CO_2_ reduction and acetaldehyde degradation, SPP treatment of TiO_2_(AB) resulted in very active photo‐ and photothermal catalyst functioning upon exposure to both solar and visible light.

Change of color observed for TiO_2_(AB) after SPP treatment (Figure 1b) can be more accurately characterized by UV–vis absorption spectra. Unlike T‐0 h, absorption spectrum of T‐2 h demonstrated broad absorption in the near‐infrared region (**Figure** **3**a). As is well reported, energy level of Ov in TiO_2_ is deep and located 0.75–1.18 eV below the conduction band. We believe that this broad band in the near‐infrared region observed in UV–vis spectrum of T‐2 h originated from the freshly formed levels in the bandgap by hydrogen doping. Moreover, this hydrogen doping introduces continuous electron transition energy levels after SPP. UV–vis spectrum of T‐2 h was also blueshifted relative to the spectrum of T‐0 h, which probably occurred because of improved crystallinity after SPP treatment. Instantaneous exposure to high temperature of plasma‐in‐liquid can help to quickly achieve localized crystallization of TiO_2_(AB). To confirm this hypothesis, we used HRTEM to analyze unannealed TiO_2_(AB) nanoparticles with weak crystallization, which were exposed to SPP for 8 h (Figure S22, Supporting Information). Lattice fringes and FFT images of unannealed TiO_2_(AB) before SPP were not very clear (Figure S22a, Supporting Information), which indicates weak crystallization.^[^ ^26^ ^]^ HRTEM of unannealed TiO_2_(AB) treated by SPP for 8 h demonstrated much clear lattice fringes and sharp FFT images (Figure S22b, Supporting Information), which confirmed that SPP treatment indeed improved crystallinity of TiO_2_(AB) and annihilate some Ov. To further confirm, we also collect the Raman spectra of the weak crystallized TiO_2_ before and after solution plasma treatment (Figure S23, Supporting Information). The Raman bands of weak crystallized TiO_2_ exhibit stronger signal after solution plasma treatment, indicating the improved crystallinity by solution plasma. Thus, SPP treatment is proven effective for slight decrease of Ov content and introduction of shallow energy level via hydrogen doping.

**Figure 3:**
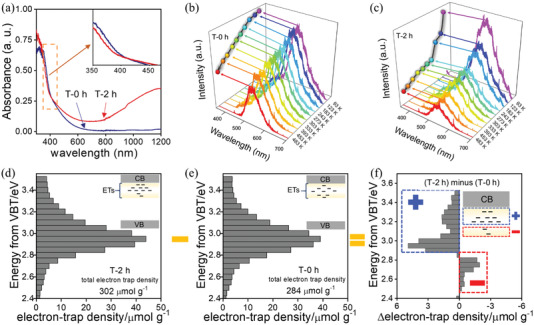
a) Ultraviolet–visible absorption spectra of TiO_2_(AB) treated with SPP for 0 and 2 h (samples T‐0 h and T‐2 h, respectively). Insert shows enlarged 350–450 nm region. Temperature‐dependent photoluminescence of b) T‐0 h and c) T‐2 h. Reversed double‐beam photoacoustic spectroscopy of d) T‐2 h and e) T‐0 h. Numbers shown on the graphs in “<>” denote total density of the trapped electrons (in µmol g^−1^); trapped electrons: ETs. f) Reversed double‐beam photoacoustic spectroscopy of Δelectron‐trap‐density between T‐2 h and T‐0 h samples.

Temperature dependent photoluminescence (PL) was used to study distribution of defects.^[^ ^50^, ^51^, ^52^ ^]^ Typically, PL intensity directly correlates with location of defects in TiO_2_. Contributions of different defects to PL spectra can be clearly seen at elevated temperatures. Difference in PL intensity at any given temperature depends on competition between charge separation at shallow energy state and charge combination at deep energy states. Thus, as temperature increases, electrons trapped by shallow energy state of hydrogen dopant tend to migrate to the surface, and electrons trapped by deep energy state of Ov recombine with holes.^[^ ^53^, ^54^ ^]^ PL spectrum of T‐0 h showed gradual intensity decrease as temperature increased from 93 to 483 K, which indicates that hydrogen dopant predominated over Ov (Figure 3b). T‐2 h showed a drastic decrease of PL intensity from 93 to 243 K and a more gradual decrease from 243 to 483 K, implying an obvious change of defect distribution (Figure 3c). This sudden PL intensity decrease reflects high content of hydrogen dopant in T‐2 h especially in comparison to T‐0 h.

Reversed double‐beam photoacoustic spectroscopy (RDB‐PAS) was conducted to further understand defect distribution in TiO_2_(AB). RDB‐PAS can measure energy‐resolved distribution of electron traps (ERDT) and provide valuable information on their density and content.^[^ ^55^, ^56^, ^57^ ^]^ Electron trap density during catalytic reaction over T‐2 h was higher than over T‐0 h (Figure 3d,e). This implies that more electron traps existed in T‐2 h than in T‐0 h. Differential electron trap density (Δelectron‐trap‐density, calculated by subtracting value for T‐0 h from the value for T‐2 h) provides additional information on defect distribution along different energy levels in TiO_2_(AB). Positive Δelectron‐trap‐density was observed at shallow levels near the conduction band (Figure 3f). Since hydrogen dopant is typically located at shallow levels, positive value of Δelectron‐trap‐density strongly supports introduction of hydrogen dopant after SPP treatment. Negative Δelectron‐trap‐density is located at deep levels far from the conduction band. Ov are located at deep levels, and negative Δelectron‐trap‐density strongly supports lower content of Ov after SPP treatment. The darkening of TiO_2_(AB) after SPP treatment is induced by introduction of hydrogen dopant. As shown in Figure 3a, the absorption spectrum of darker TiO_2_(AB), T‐2 h, display broad absorption in the near‐infrared region, suggesting SPP can introduce additional intermediate level between conduction band and oxygen vacancy of TiO_2_. Meanwhile, the reversed double‐beam photoacoustic spectroscopy (RDB‐PAS) shown in Figure 3f reveals the introduction of shallow level near the conduction band. The combined analysis demonstrates that the darkening of TiO_2_(AB) originates from hydrogen doping.

Behavior of photogenerated charges during catalytic reactions involving different TiO_2_ materials was analyzed using transient surface photovoltage (TPV) technique. This technique can provide insight on dynamic properties of photoinduced charge carriers in semiconductors, including generation, separation, and recombination of photogenerated charges.^[^ ^58^, ^59^ ^]^ TPV spectra showed stronger photovoltaic response from the system containing T‐2 h than from the T‐0 h (Figure S24, Supporting Information). Time to achieve maximum photovoltage value in the system containing T‐2 h was longer than for T‐0 h, which demonstrates better charge separation ability of TiO_2_(AB) catalyst treated by SPP. Such behavior is very beneficial for catalytic activity enhancement upon exposure of the system to solar light.

All the results mentioned above support our hypothesis that SPP treatment caused defect redistribution, which significantly enhanced TiO_2_(AB) catalytic activity under solar irradiation. TiO_2_(AB) treated by SPP demonstrated strong visible light absorption and high charge separation capability, which are also very beneficial for improving TiO_2_(AB) catalytic ability. These two phenomena should equally enhance TiO_2_(AB) activity during photo‐ and photothermal catalytic reactions. However, higher activity over SPP modified TiO_2_(AB) was observed for photothermal catalytic reactions than for photocatalytic ones. Better performance in photothermal reactions is very likely because of electrons near shallow energy state released at high temperatures. The higher content of shallow energy state, the easier it is for the electrons to be released from their traps. All these freshly freed electrons accelerate surface reduction of adsorbed species.

Thermal oxidation of acetaldehyde in the dark (Figure S19, Supporting Information) implies very strong oxidation ability of lattice oxygen, which is also induced by Ov presence. To verify this assumption, we recorded oxygen TPD spectra (Figure S25, Supporting Information). Thermal escape of lattice oxygen observed for the T‐2 h was detected at lower temperature than for the T‐0 h. Stronger signal from T‐2 h indicates higher amount of escaped lattice oxygen. Thus, T‐2 h material possessed lattice oxygen with lower activity than T‐0 h. H doping in T‐2 h makes it very prone to oxidative half‐reactions, which perform better in photothermal catalysis.^[^ ^60^, ^61^ ^]^ As a result, such excellent photothermal catalytic activity of T‐2 h can be attributed to the synergy between strong visible light absorption, high charge separation capability, thermal release of trapped electrons and thermal oxidation of the absorbed species by lattice oxygen.

Schematic of the mechanism of enhanced catalytic activity of SPP‐treated TiO_2_(AB) under solar light is shown in **Figure** **4**. As‐synthesized TiO_2_(AB) contained deep energy states of oxygen vacancies. In this defected phase, photogenerated electrons transition either from the valence band to the conduction band under UV light or to the defect level of oxygen vacancies under visible light. However, oxygen vacancies tend to recombine photogenerated electrons and holes. Despite the strong visible light absorption, serious charge recombination at Ov limits TiO_2_ photo‐ and photothermal catalytic activity. After SPP, TiO_2_(AB) contained less Ov and newly formed hydrogen dopant. Such redistribution of defects results in formation of continuous energy levels below the conduction band, which trap more electrons, promoting charge separation and supporting visible light absorption. At the same time, activation of lattice oxygen is accelerated, and release of electrons trapped by Ov is enhanced. All these processes significantly enhance photo‐ and photothermal catalytic activity under solar light.

**Figure 4:**
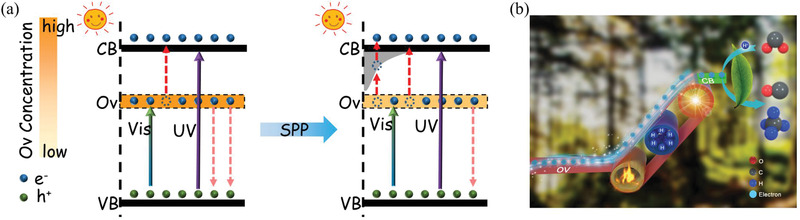
a) Schematic illustration of the electron–hole separation mechanism for T‐0 h and T‐2 h samples during photocatalysis driven by UV and visible light irradiation. b) Schematic illustration of electron transfer from Ov to CB under solar light irradiation with thermal assist.

We also studied discharge time and Ov content in TiO_2_(AB) to understand how solution plasma interacts with TiO_2_(AB). To study how SPP discharge time affects final TiO_2_ properties, as‐synthesized T‐0 h was SPP‐treated for 4 h (named T‐4 h). XRD, Raman, HRTEM, and XPS analyses showed no obvious changes in phase composition as SPP‐treatment time increased (Figures S26 and S27, Supporting Information). UV–vis spectra showed weaker adsorption for T‐4 h in the visible to near‐infrared spectrum region in comparison with the T‐2 h (**Figure** **5**a). However, degree of blueshift of intrinsic absorption is enhanced. RDB‐PAS showed smaller electron trap density as SPP treatment time increased: densities for T‐2 h and T‐4 h were 310 and 281 µmol g^−1^, respectively (Figure S28, Supporting Information). Δelectron‐trap‐density between T‐4 h and T‐2 h demonstrated that hydrogen dopant at the shallow level increased and Ov at the deep level decreased (Figure 5c). Δelectron‐trap‐density between T‐4 h and T‐0 h revealed similar decreasing trend at the deep level (Figure 5d) while amount of hydrogen dopant at the shallow level demonstrated slight decrease. Both T‐4 h and T‐2 h exhibited increased hydrogen dopant and decreased Ov in comparison to T‐0 h. Relative to T‐2 h, contents of hydrogen dopant and Ov in the T‐4 h were lower. ESR intensity of our SPP‐treated TiO_2_(AB) catalysts decreased gradually as SPP‐treatment time increased confirming decreased concentration of Ov (Figure S29, Supporting Information).

**Figure 5:**
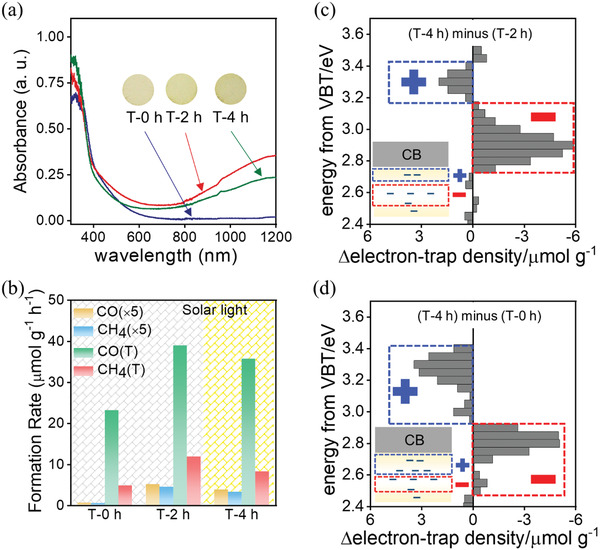
a) Ultraviolet–visible absorption spectra of TiO_2_(AB) treated by SPP for T‐0 h, T‐2 h, and T‐4 h. Circles around each sample name is a photograph of the actual sample color. b) Photocatalytic and photothermal catalytic activities of CO_2_ conversion over T‐x upon exposure to solar light. Reversed double‐beam photoacoustic spectroscopy of Δelectron‐trap‐density c) between T‐4 h and T‐2 h and d) between T‐4 h and T‐0 h.

T‐4 h showed worse performance during photothermal catalytic reduction of CO_2_ than T‐2 h judging by the amount of CO or CH_4_ formed under solar (Figure 5b) and visible light (Figure S30, Supporting Information). The same trend was observed for the photothermal catalytic acetaldehyde degradation (Figure S31, Supporting Information). Lower photothermal catalytic activity of the T‐4 h was very likely because of lower content of defects. Thus, SPP‐treatment time is the major factor responsible for defect distribution between the gap of Ov and conduction band of TiO_2_(AB). The optimal SPP treatment time for hydrogen doping can be understood by a balance between elimination of defects at deep level and introduction of hydrogen dopants at shallow level of Ov‐TiO_2_ in solution plasma process. The solution plasma process is accompanied by instant high temperature and flux of hydrogen radical. The instant high temperature is beneficial for elimination of defects, which is verified by improved crystallinity of SPP treated weaker crystallized TiO_2_ (Figures S22 and S23, Supporting Information). In addition, the electron‐trap‐density determined by RDB‐PAS is decreased from 302 µmol g^−1^ over T‐2 h to 281 µmol g^−1^ over T‐4 h. The combined results confirm the role of elimination of defects by solution plasma. The flux of hydrogen radical is beneficial for introduction of hydrogen dopant. As shown in Figure 3f, the Δelectron‐trap‐density between T‐2 h and T‐0 h reveals the introduction of shallow defect level. Furthermore, the Δelectron‐trap‐density between T‐2 h and T‐4 h shown in Figure 5c reveals the diminishing of defects at shallow level with the prolonging of solution plasma treatment. Therefore, the optimal SPP treatment time of 2 h can be explained by balance between elimination of defects at deep level and introduction of hydrogen dopants at shallow level of Ov‐TiO_2_ in solution plasma process.

To prepare single phase Ov‐TiO_2_, a pure anatase phase was obtained by annealing at 450 °C, while other conditions kept the same as TiO_2_(AB). The solution plasma treatment on anatase TiO_2_ was lasted for 2 h, which is the same as that TiO_2_(AB). For simplicity, the as‐calcined sample at 450 °C is named as 450–0 h. The 450–0 h sample subjected to solution plasma treatment for 2 h is named as 450–2 h. HRTEM, XRD, and Raman analysis (Figure S32, Supporting Information) confirm the single phase of anatase and solution plasma treatment does not brings significant structural change of TiO_2_, which is similarly revealed by aforementioned study on solution plasma treated TiO_2_(AB). XPS comparison (Figure S33, Supporting Information) on 450–0 h and 450–2 h exhibits hardly observed shift of binding energy, further confirming the insignificant structural change. ESR analysis (Figure S34, Supporting Information) demonstrates a slight decrease of signal intensity related to oxygen vacancy after solution plasma treatment, which is in consistence with the regular on solution plasma treated TiO_2_(AB). The photothermal catalytic results toward reduction of CO_2_ over 450–0 h and 450–2 h under solar light are shown in Figure S35 (Supporting Information). The evolution rate of CO and CH_4_ over 450–2 h is 1.40 and 1.44 times higher than 450–0 h, respectively. In other word, the solution plasma treatment effectively promotes CO_2_ reduction over anatase TiO_2_. Accordingly, besides TiO_2_(AB), the solution plasma also works well in activation of TiO_2_ with single anatase phase for photothermal catalytic reaction of CO_2_.

To demonstrate applicability of SPP treatment to other semiconductor oxides for photo‐ and photothermal catalysis, we treated ZnO, WO_3_, and Ta_2_O_5_. Their extensive characterization demonstrated no changes in their phase compositions (Figures S36 and S37, Supporting Information) and no noticeable shifts of the intrinsic band edges (Figure S38, Supporting Information) after SPP treatment. Photo‐ and photothermal degradation of acetaldehyde using these SPP‐treated oxides as catalysts demonstrated enhanced activity for all of them (Figure S39, Supporting Information). Thus, SPP can be universally applied to activate and to enhance catalytic properties of variety of semiconductor oxides.

In summary, we developed a strategy to activate TiO_2_ to be used as a catalyst for solar‐induced photo‐ and photothermal degradation of acetaldehyde and CO_2_ reduction. This strategy involved synthesis of TiO_2_ with rich oxygen vacancies using hydrothermal method followed by hydrogen doping in SPP. UV–vis absorption, temperature‐dependent PL, RDB‐PAS, and ESR spectra demonstrated that SPP increased hydrogen dopant content and decreased content of oxygen vacancies. Moreover, SPP technology does not cause any structural or chemical changes and allows initial material to maintain its strong visible light absorption. TPV spectra demonstrated enhanced charge separation of the resulting TiO_2_ after redistribution of defects. Because of this enhanced charge separation and visible light absorption, catalytic activity was significantly improved. It was confirmed by the catalytic acetaldehyde degradation and CO_2_ reduction under visible and solar light. This was also demonstrated for both photo‐ and photothermal‐catalytic reactions. For the best SPP effect, initial material needs to be rich with oxygen vacancies. We demonstrated applicability of SPP treatment to variety of semiconductor oxides used as catalysts for solar‐induced photo‐ and photothermal reactions.

## Experimental Section

##### Synthesis of TiO_2_ (AB)

A portion of 0.8 mL TiCl_4_ was added into 45 mL of ice water and stirred until a clear colorless solution was obtained. Then, 5 mL of 25% NH_3_·H_2_O, 60 mL of deionized water, and 10 mL of 30% H_2_O_2_ were added and mixed until a clear yellow solution formed. A portion of 0.5 g glycolic acid was added to this yellow solution, which was then heated at 80 °C for 390 min to remove excess H_2_O_2_ and NH_3_. The resulting yellowish gel was mixed with 50 mL of deionized water. pH value of the resulting yellow suspension was adjusted to 2.0 by H_2_SO_4_. The suspension was transferred into two 50 mL autoclaves and heated at 160 °C for 50 min. After the hydrothermal reaction completion, the resulting product was centrifuged, washed several times with ethanol and deionized water, dried at 80 °C, and finally annealed at 370 °C for 1 h. The resulting material was named TiO_2_(AB), and it was composed of anatase and TiO_2_(B). The two materials were also synthesized using the same procedure but with a 450 °C final calcination step and without any calcination. The sample of 450 °C is a monophase of anatase. The sample of without any calcination is a weak crystallized TiO_2_.

##### Solution Plasma Process

SPP was performed as described in detail elsewhere.^[^ ^39^ ^]^ A 0.4 g portion of each material was placed into a reactor containing 200 mL of KCl solution with 300 µS cm^−1^ electrical conductivity. A tungsten rod, 3 mm in diameter, and a tungsten tube (with 3 and 1 mm outer and inner diameters, respectively) were used as electrodes. Each electrode was inserted into a silicone plug and placed in the middle part of the reactor. The distance between two electrode tips was < 0.5 mm. N_2_ was introduced into the reactor through the hollow tungsten electrode. Plasma was generated by a bipolar‐DC pulsed power supply (Kurita, Japan). The pulse width and frequency were 2.0 µs and 20 kHz, respectively. The temperature of solution (which was 10 °C) was controlled by a water chiller. Discharge time was 2 h. Samples underwent SPP treatment were marked as T‐x, where “T” stands for TiO_2_(AB) and “x” represents discharge time. All samples were dried at 60 °C.

## Conflict of Interest

The authors declare no conflict of interest.

## Supporting information

Supporting Information
