# Supplementary material for: Enhanced Solar Photothermal Catalysis over Solution Plasma Activated TiO2
Source: Adv Sci (Weinh). 2020 Jun 11;7(16):2000204. doi: 10.1002/advs.202000204 (PMC7435248; doi:10.1002/advs.202000204)
Supplement: Supplementary file 1 — Supporting Information [file ADVS-7-2000204-s001.pdf]

WILEY-VCH

Copyright WILEY-VCH Verlag GmbH & Co. KGaA, 69469 Weinheim, Germany, 2020.

Supporting Information

Enhanced Solar Photothermal Catalysis over Solution Plasma Activated TiO_2

Fei Yu, Changhua Wang, Yingying Li, He Ma, Rui Wang, Yichun Liu, Norihiro Suzuki,*

*Chiaki Terashima, Bunsho Ohtani, Tsuyoshi Ochiai, Akira Fujishima, Xintong Zhang**

Dr. F. Yu, Prof. C. Wang, Dr. Y. Li, Dr. H. Ma; R. Wang, Prof. Y. Liu, Prof. X. Zhang
Key Laboratory of UV-Emitting Materials and Technology of Chinese Ministry of Education
Northeast Normal University
Changchun 130024, China
E-mail: wangch100@nenu.edu.cn; xtzhang@nenu.edu.cn

Prof. N. Suzuki, Prof. C. Terashima, Prof. A. Fujishima
Photocatalysis International Research Center
Research Institute for Science & Technology
Tokyo University of Science,
2641 Yamazaki, Noda, Chiba 278-8510, Japan

Prof. B. Ohtani
Graduate School of Environmental Science
Hokkaido University
Sapporo 060-0810, Japan

Prof. T. Ochiai
Photocatalyst Group
Research and Development Department
Local Independent Administrative Agency Kanagawa Institute of industrial Science and Technology (KISTEC)
Kanagawa 213-0012, Japan

Table of Contents

1. Material Characterization. 4

2. Catalytic Tests. 5

2.1. Photocatalytic and photothermalcatalytic reduction of CO_2. 5

2.2. Catalytic degradation of gaseous acetaldehyde. 6

2.2.1 The equation for AQE calculation. 6

3. Calculation method. 7

4. Supplementary Figures. 8

4.1 The Effects of SPP for TiO_2(AB). 8

Figure S1. XRD patterns and Raman spectra of T-0 h and T-2 h. 8

Figure S2. N_2 adsorption-desorption analysis of T-0 h and T-2 h. 9

Figure S3. ESR spectra of T-0 h and T-2 h. 10

Figure S4. XPS specrea of T-0 h and T-2 h. 11

Figure S5. Other products in photothermal catalytic CO_2 conversion. 12

Figure S6. Mass spectra of ^{13}CO over T-2h. 13

Figure S7. Recycling test of T-2 h catalyst via photothermal of CO_2 reduction. 14

Figure S8. XRD patterns and Raman spectra after catalytic test. 15

Figure S9. XPS spectra after catalytic test. 16

Figure S10. ESR spectra after catalytic test. 17

Figure S11. The surface top view of TiO_2(101). 18

Figure S12. Overlooking and side view of TiO_2(101) surface. 18

Figure S13. The top view and side view after optimization of H-doped TiO_2. 19

Figure S14. Free energy diagram of PDS and the corresponding Ea. 20

Figure S15. Acetaldehyde degradation test under UV, Biue and Green light. 21

Figure S16. Acetaldehyde degradation over T-0 h, T-2 h and commercial P25. 22

Figure S17. ESR spectra of T-2 h under light and in dark. 23

Figure S18. Infrared thermal images of T-2 h and T-0 h. 24

Figure S19. Acetaldehyde degradation under dark at 70℃. 25

Figure S20. Cycling test of Photocatalytic acetaldehyde degradation. 26

Figure S21. The AQE of Photocatalytic acetaldehyde degradation. 27

Figure S22. HRTEM images of WCT-0 h and WCT-8 h. 28

Figure S23. XRD images and Raman spectra of WCT-0 h and WCT-8 h. 29

Figure S24. TPV spectroscopy of T-0 h and T-2 h. 30

Figure S25. O_2-TPD profiles of T-0 h and T-2 h. 31

4.2 The impact of processing time for TiO_2(AB) treated by SPP. 32

Figure S26. TEM, HRTEM images, XRD patterns and Raman of T-x. 32

Figure S27. XPS specrea of T-x. 33

Figure S28. Reversed double-beam photoacoustic spectroscopy of T-4 h. 34

Figure S29. ESR spectra of T-x. 35

Figure S30. PC and PTC of CO_2 reduction over T-x under visible light. 36

Figure S31. PC and PTC of acetaldehyde degradation over T-x. 37

4.3 The Effects of SPP for other TiO_2 38

Figure S32. HRTEM images, XRD patterns and Raman of 450-x. 38

Figure S33. XPS spectra of 450-x. 39

Figure S34. ESR spectra of 450-x. 40

Figure S35. PTC of CO_2 reduction over 450-x. 41

4.4 The Effects of SPP for other semiconductor oxides. 42

Figure S36. XRD patterns of different semiconductor oxide samples untreated or treated by SPP. 42

Figure S37. Raman spectra of different semiconductor oxide samples untreated or treated by SPP. 43

Figure S38. The UV-vis absorption spectra of different semiconductor oxide samples untreated or treated by SPP. **44**

Figure S39. Time curves of acetaldehyde degradation and CO_2 generation over different semiconductor oxide samples untreated or treated by SPP. **45**

Table S1. Comparison of photocatalytic activity of CO_2 reduction over TiO_2-based catalysts. **46**

Table S2. Compared the relationship between products and reaction conditions in photocatalytic CO_2 reduction. **47**

5. References **48**

1. Material Characterization.

UV-vis diffuse reflectance (DR) spectra were collected on a PerkinElmer UV Win Lab spectrophotometer, and $BaSO_4$ was used as a reference. The crystal structure and phase identification were characterized by X-ray diffraction (XRD, Rigaku, D/max-2500 X-ray diffractometer) using Cu Kα radiation (λ = 1.5406Å). The PL and Raman spectra were recorded with a Horiba Evolution Spectrophotometer System which under an excitation of a 325 nm and 488 nm Ar^+ laser. The morphology of the samples was characterized with a transmission electron microscope (TEM, a JEM-2100 transmission electron microscope) at an acceleration voltage of 200 kV. X-ray photoelectron spectroscopy (XPS) experiments were performed on a Thermo SCIENTIFIC ESCALAB 250Xi instrument with an Al Kα ADES (hν =1486.8eV). Electron spin resonance (ESR) spectra were performed by a Bruker EMXnano spectrometer. Spectra were recorded at 110K and acquired with a modulation frequency of 9.63 GHz, and microwave powers of 1.259 mW. O_2-Temperature-programmed desorption (TPD) measurement was performed on a Quantachrome TPRWin v3.52 instrument. 100 mg sample was filled in an adsorption vessel. Then, the sample heated to 800 °C at a rate of 15 °C/min. The desorbed oxygen was monitored by thermal conductivity detector. ^1H NMR spectra were recorded on a Bruker AV 400 NMR spectrometer.

The procedure for measurement of ERDT according to the method developed by Ohtani et al[1-3] was briefly described as following: A stainless-steel sample holder was filled with sample and set in a photoacoustic spectroscopy (PAS) cell equipped with an electret condenser microphone and a Pylex window on the upper side. For PAS measurements, the PAS cell was filled with nitrogen, and a light beam from a xenon lamp (Spectral Products ASB-XE-175) with a grating monochromator (Spectral Products CM110 1/8m) modulated at 80 Hz by a light chopper (NF Corporation 5584A) was irradiated from 650 nm to 350 nm through the cell window to detect the PAS signal using a digital lock-in amplifier (NF Corporation LI5630), and then photoacoustic (PA) spectra were recorded to a PA spectrum of

graphite. The conduction-band bottom, as energy from the top of the valence band (VBT), of conduction-band bottom samples was estimated by the onset wavelength corresponding to the bandgap of samples in units of eV. For RDB-PAS measurements, the PAS cell was filled with methanol-saturated argon, and two light beams were introduced simultaneously using a UV quartz combiner light guide (Moritex MWS5-1000S-UV3). One was a 625-nm light beam from an LED modulated by a digital function generator (NF Corporation DF1906) at 80 Hz, and the other was continuous monochromatic light from a monochromator with a wavelength scanning from 650 nm to 350 nm with 5-nm steps. The raw spectrum obtained was differentiated from the lower-energy side and calibrated with the reported total electron-trap density in units of μmol g^{-1} measured by a photochemical method to obtain an ERDT pattern.

2. Catalytic Tests

2.1. Photocatalytic and photothermalcatalytic reduction of CO_2

Catalytic reduction of CO_2 with H_2O was conducted in a stainless autoclave reactor (100 mL) with a quartz window on the top. Solar light and visible light were provided by a Hayashi LA 410 Xenon lamp (150 W). The solar light intensity was 100 mW cm^{-2}. Visible light was acquired with a 420 nm long-pass filter with light intensity of 100 mW cm^{-2}. In the photocatalytic test, 20 mg of catalyst was ultrasonically dispersed in 1 mL of deionized water and placed in the reactor. Then, the autoclave was sealed, and the internal air was degassed quickly and completely using high-purity CO_2 (99.999%) for 20 mins at room temperature and atmosphere pressure. In the photothermalcatalytic test, the reactor was heated to 120 ^oC, while other conditions kept the same as photocatalytic test. The gaseous mixture was qualitatively and quantitatively analyzed using a Shimadzu 2014C GC instrument. The isotopic experiment was carried out to check the product of CO_2 reduction using $^{13}CO_2$ (Aldrich Comp. 99%) as reaction gas. The experiment was performed on gas

chromatography-mass spectrometry (Agilent 6890 GC/59973MSD (EI), HP-MOLESIEVE equipped with Micropacked column).

2.2. Catalytic degradation of gaseous acetaldehyde

The catalytic degradation was performed in 500 mL Pyrex glass vessel under solar light, visible light and monochromatic light (blue and green), respectively. UV light and visible light were provided by a Hayashi LA -410 Xenon lamp (150W) that the UV light intensity was 3 mW cm^{-2}. Visible light was acquired with a 450 nm long-pass filter which light intensity was 100 mW cm^{-2}. The AQE was measured under the single pass filter with 365 nm, 400 nm, 450 nm and 500 nm, the light intensity was 1 mW cm^{-2}. The blue and green light were acquired by LED and both the light intensity was 200 mW/cm^2. In the photocatalytic test, 0.05 g of TiO_2 catalysts were spread over a square of 2.5 cm×2.5 cm glass sheet with 0.2 mL deionized water and dried at 60 °C in the oven. The glass sheet was pretreated by ultrasonic cleaning in ethanol and deionized water for several times, and irradiation under ozone-ultraviolet for 30 min. The coated glass was put into glass vessel and then high purity air was filled into glass vessel. 5 μmol (200 ppm) of acetaldehyde gas was injected into the reactor and dark adsorption for 30 min. The concentration of acetaldehyde and CO_2 were measured on gas chromatograph (SP-2100A, BFRL Co.). In the photothermalcatalytic test, the reactor was heated to 343 K, while other conditions were kept the same as that in photocatalytic test.

2.2.1 The equation for AQE calculation

AQE = (N_e×number of produced molecule)/(number of incident photons) (1)

Number of incident photons = (light Intensity×A)/(Single electron energy) (2)

Single electron energy = $h\ c/\lambda$ (3)

N_e × number of produced molecule= xn×NA (4)

A: lighting area $h=6.626\times10^{-34}$ J·s $c=3\times10^{8}$ m/s

λ: Incident wavelength (365 nm, 400 nm, 450 nm and 500 nm)

x: the number of electron transfer   n: amount of substance

3. Calculation method

All the DFT calculations were performed by Vienna Ab initio Simulation Package (VASP) [4] with the projector augmented wave (PAW) method[5] and the generalized gradient approximation (GGA) of Perdew-Burke-Ernzerhof (PBE)[6] functional. The energy cutoff for the plane wave basis set was set as 450 eV. The force on each atom less than 0.03 eV/ Å was adopted as convergence criterion during the geometry optimization. The self-consistent calculations apply a convergence energy threshold of 10^{-4} eV. The supercell was constructed by a three-layer 3×1 TiO_2(101) slab with 108 atoms and a 15 Å vacuum, in order to avoid the interaction between periodic structures. The 2×2×1 Monkhorst and Pack[7] k-point sampling was used.

The free energies of the CO_2 reduction steps (CRR) were calculated by the equation:[8] $\Delta G = \Delta E_{DFT} + \Delta E_{ZPE} - T\Delta S$, where ΔE_{DFT} is the DFT electronic energy difference of each step, ΔE_{ZPE} and ΔS are the correction of zero-point energy and the variation of entropy, respectively, which are obtained by vibration analysis, T is the temperature (T = 300 K). According to previous study,[8,9] corrections of -0.51, -0.08 and +0.13 eV have been used to eliminate PBE error for gas-phase CO, H_2 and CO_2, respectively.The climbing-image nudged elastic band (CI-NEB) method implemented in VASP was used to search the saddle points and minimum energy pathways.

4. Supplementary Figures

4.1 The Effects of SPP for TiO_2(AB)

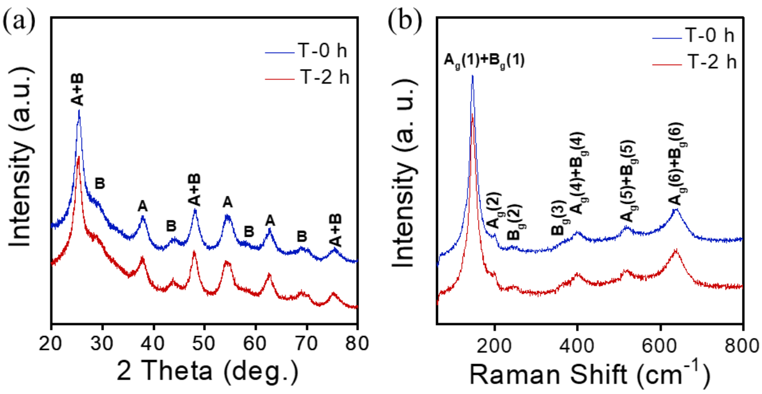

Figure S1. (a) XRD patterns and (b) Raman spectra of TiO_2(AB) treated by SPP for 0 and 2 hours.

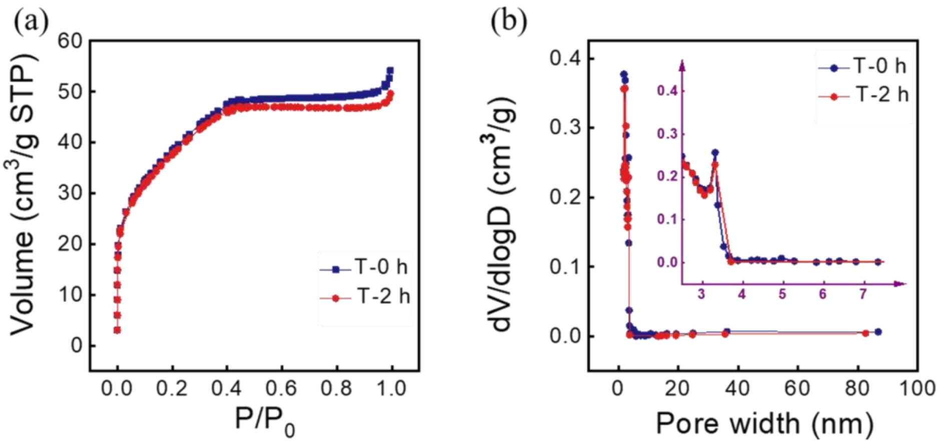

Figure S2. (a) N_2 adsorption-desorption isotherms curves of T-x; (b) the corresponding pore size distribution plots of T-x, the insert is the larger apart.

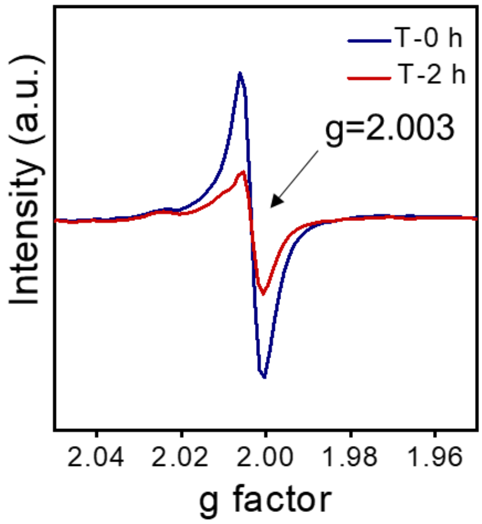

Figure S3. ESR spectra of T-0 h and T-2 h.

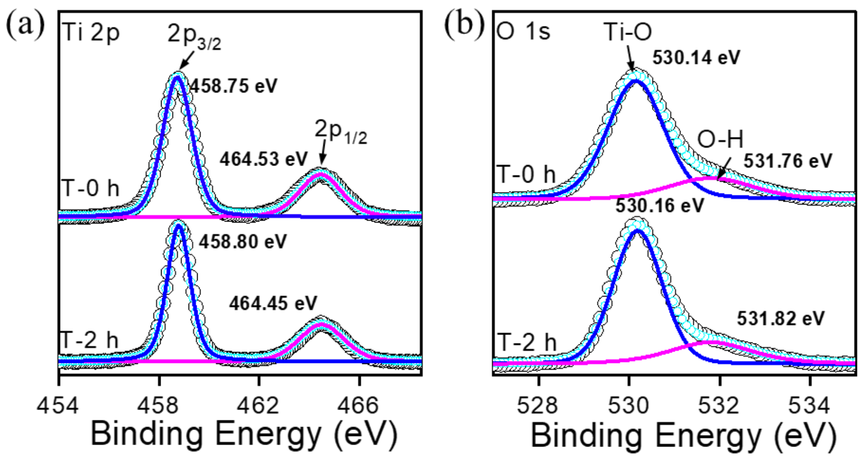

Figure S4. XPS spectra of T-0 h and T-2 h.

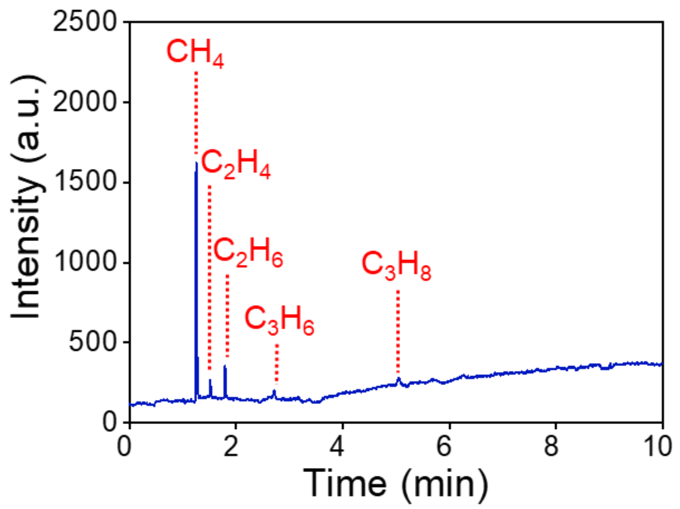

Figure S5. Other products formed in photothermal catalytic CO_2 conversion over T-2 h.

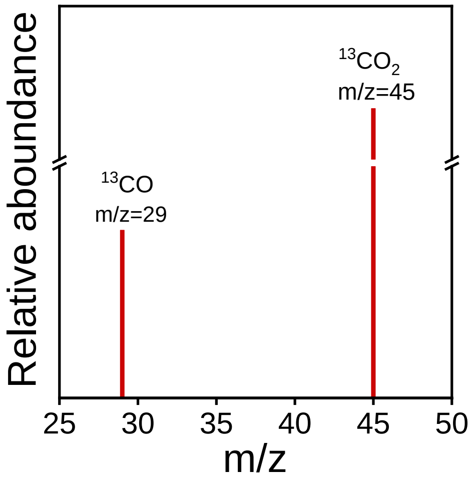

Figure S6. Mass spectra of ^{13}CO obtained by the photothermal catalytic reduction of $^{13}CO_2$ over T-2 h.

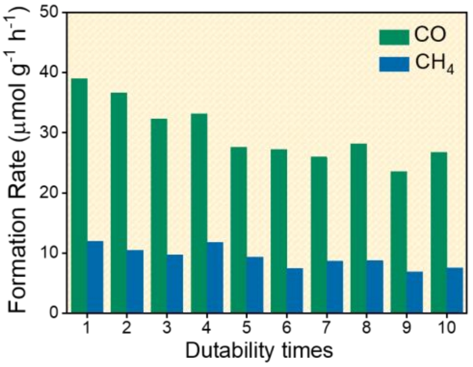

Figure S7. Recycling test of T-2 h catalyst via photothermal of CO_2 reduction.

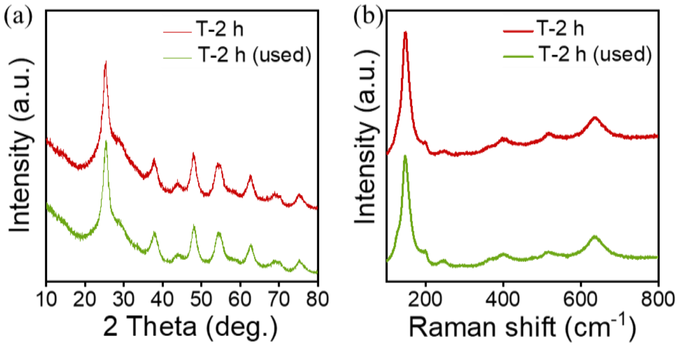

Figure S8. (a) XRD patterns and (b) Raman spectra of the T-2 h and the used T-2 h after catalytic test.

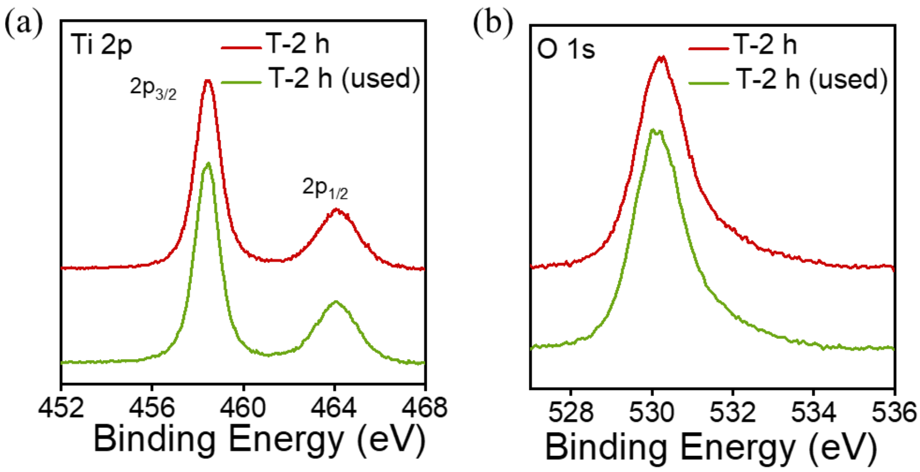

Figure S9. (a) Ti 2p and (b) O 1s XPS spectra of the T-2 h and the used T-2 h.

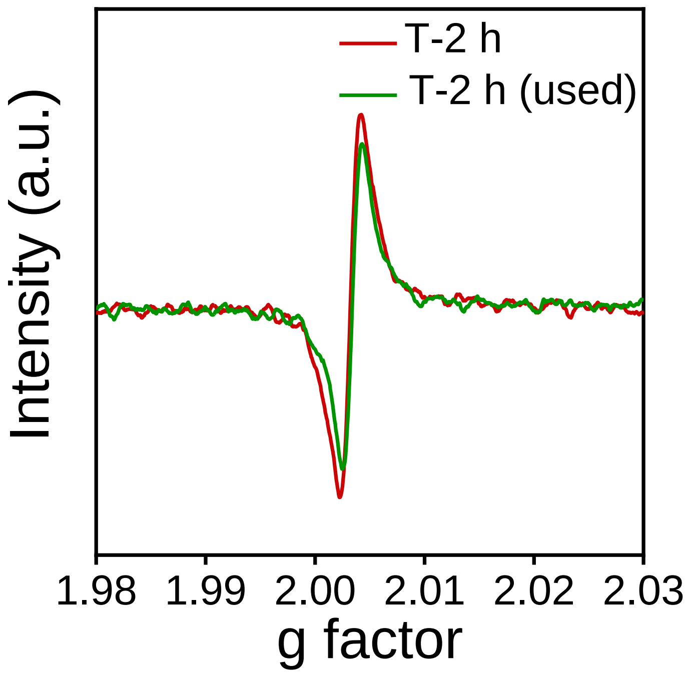

Figure S10. ESR spectra of the T-2 h and the used T-2 h.

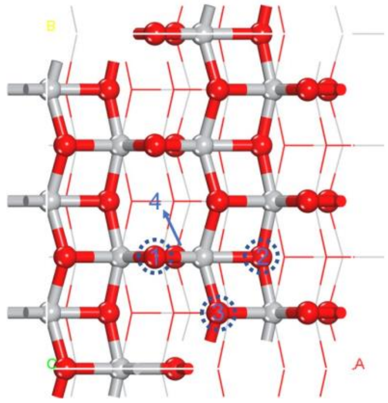

Figure S11. The surface top view of $TiO_2(101)$ and potential four surface O vacancy sites.

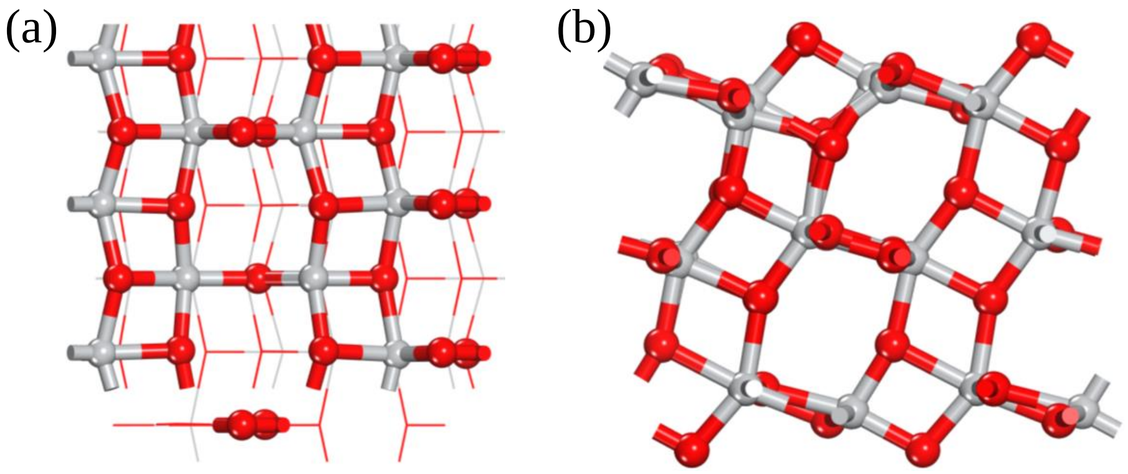

Figure S12. (a) Overlooking and (b) side view of $TiO_2(101)$ surface with site No. 1 oxygen vacancy after optimization.

First, the thermal stability of four different surface oxygen vacancies on $TiO_2(101)$ surface was tested (Figure S11).On the calculated surface, the unsaturated coordination site at No. 1 oxygen on the end surface is more likely to be detached, resulting in vacancy defect. The relative energies of the four sites were 0, +0.41, +0.97 and +0.61 eV, respectively. The optimized structure of oxygen vacancy at site No.1 is shown in Figure S12.

(a) (b)

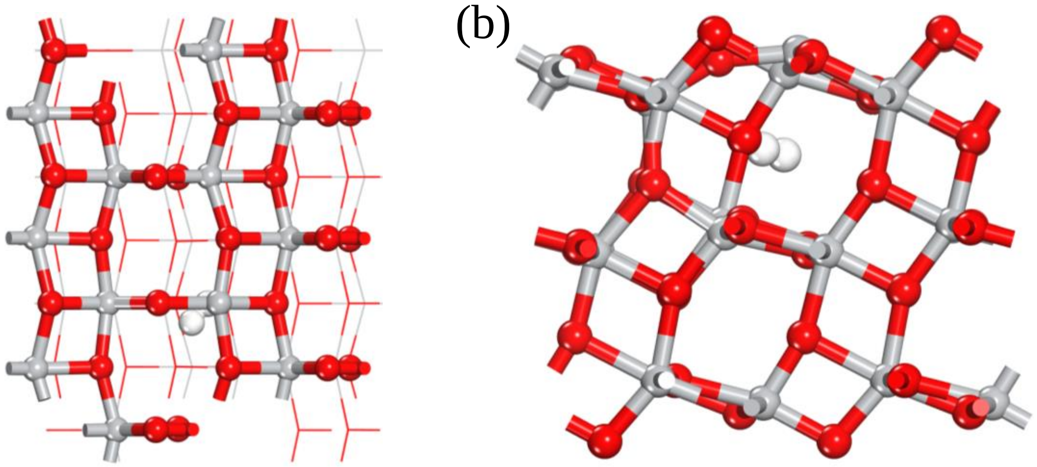

Figure S13. (a) The top view and (b) side view after optimization of H-doped TiO_2.

On the basis of finding the most stable O vacancy configuration and referring to previous work[10], we carried out the calculation of bulk hydrogen doping, and the optimized structure is shown in Figure S13. It can be clearly seen that hydrogen doping breaks the saturated Ti-O bond in the adjacent position, causing the adjacent position O to rise.

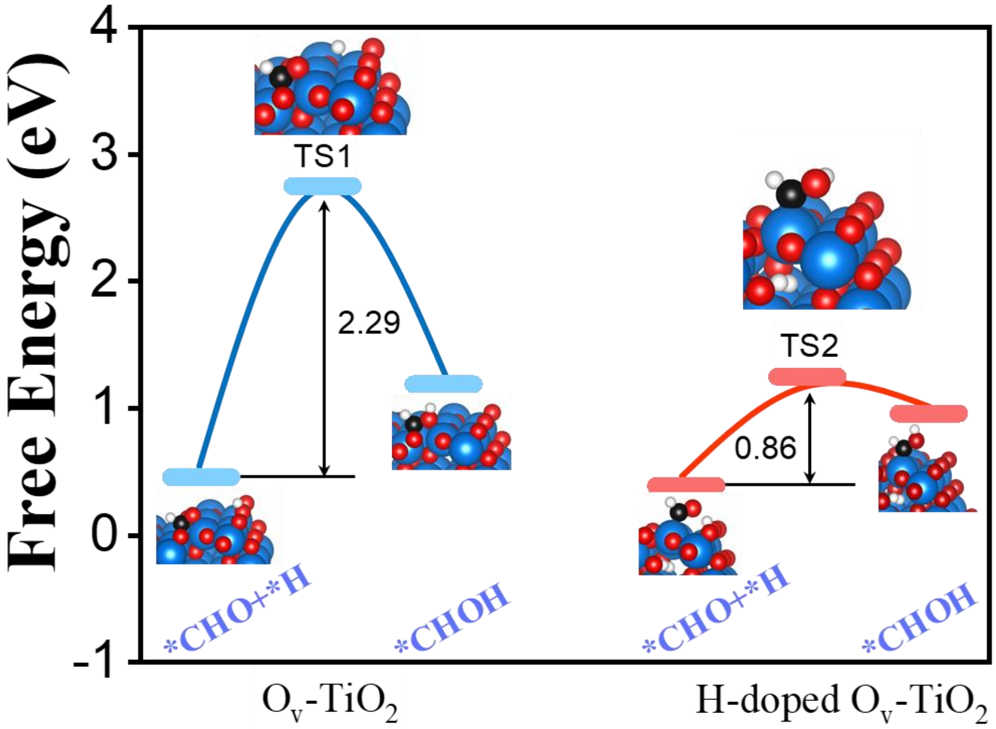

Figure S14. Free energy diagram of potential-determining step (from *CHO to *CHOH) on O_v-TiO_2 and H-doped O_v-TiO_2 and the corresponding activation energy (Ea). Insets are the corresponding structures of reaction intermediates, where the blue, red, black and white balls represent Ti, O, C and H atoms, respectively.

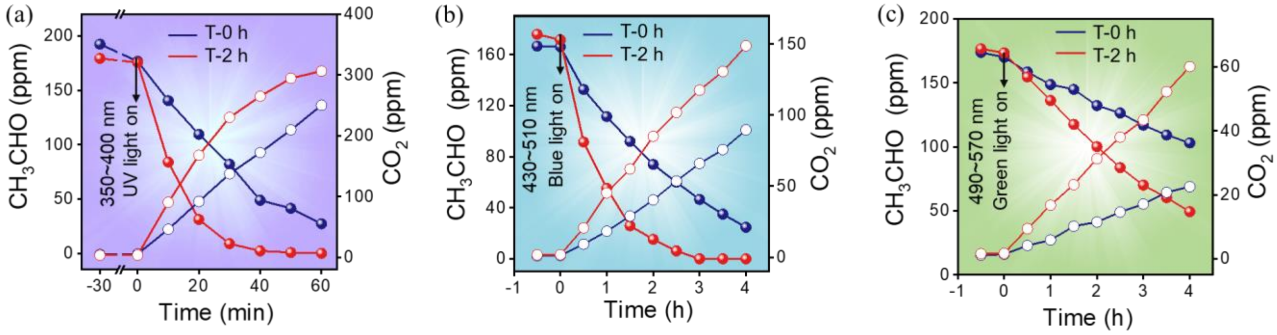

Figure S15. Acetaldehyde degradation and CO_2 reduction performed over T-0 h and T-2 h under (a) UV, (b) Blue and (c) Green light.

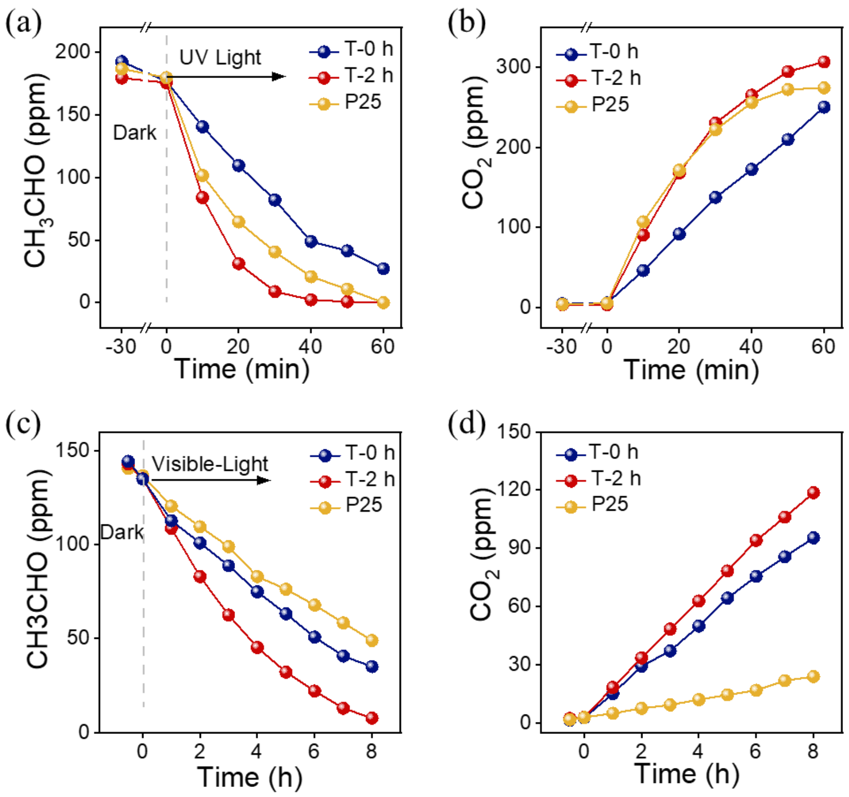

Figure S16. Acetaldehyde degradation over T-0 h, T-2 h and commercial P25: (a)-(b) under UV light and (c)-(d) under visible light.

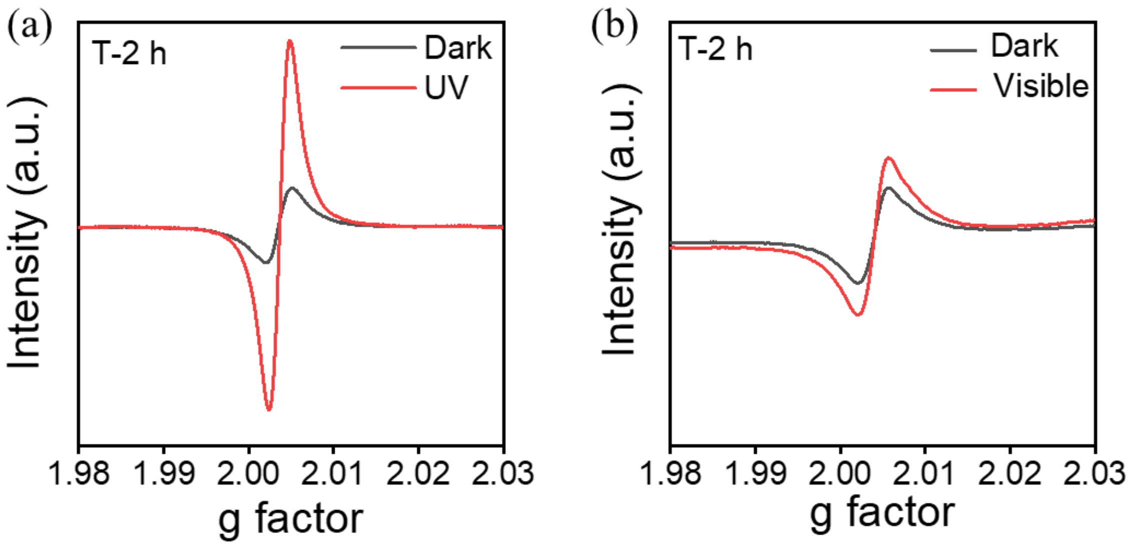

Figure S17. Compared ESR spectra of T-2 h (a) under UV light and in dark and (b) under visible light and in dark.

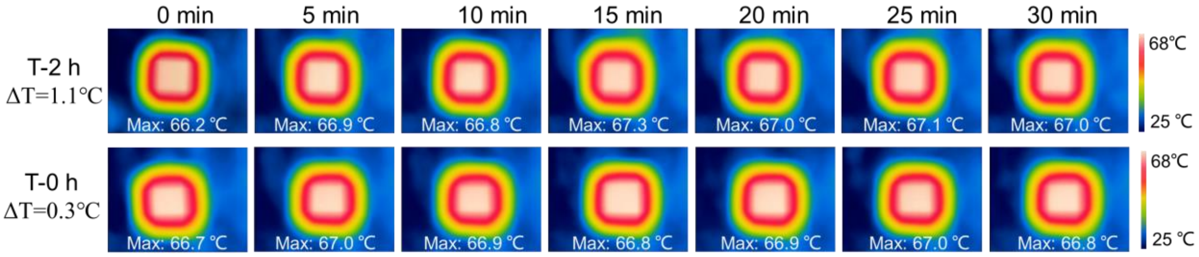

Figure S18. Infrared thermal images of T-2 h and T-0 h with the heating temperature of 70℃ under UV light irradiation.

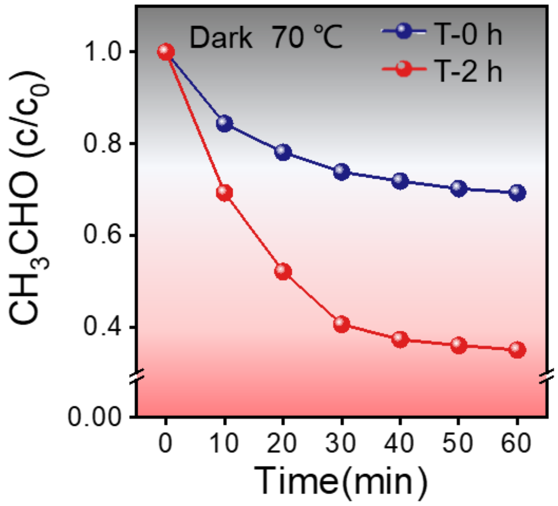

Figure S19. Acetaldehyde degradation performed over T-0 h and T-2 h under dark with heating at 70℃.

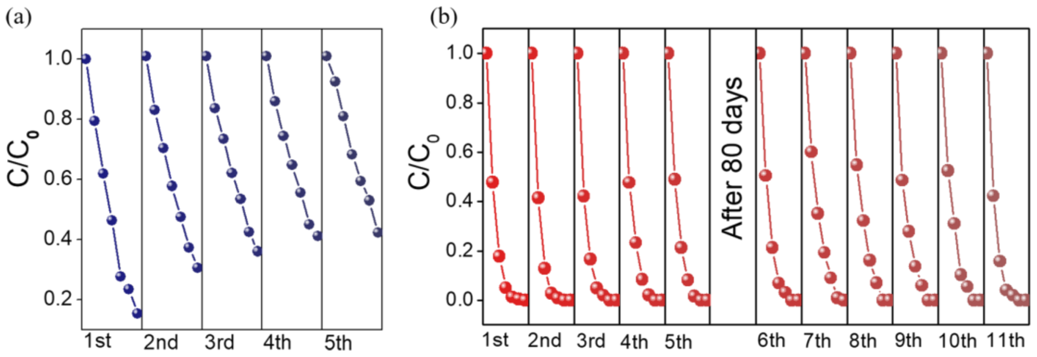

Figure S20. Cycling test of Photocatalytic degradation curve under UV light irradiation for (a) T-0 h and (b) T-2 h during the photocatalytic degradation of acetaldehyde.

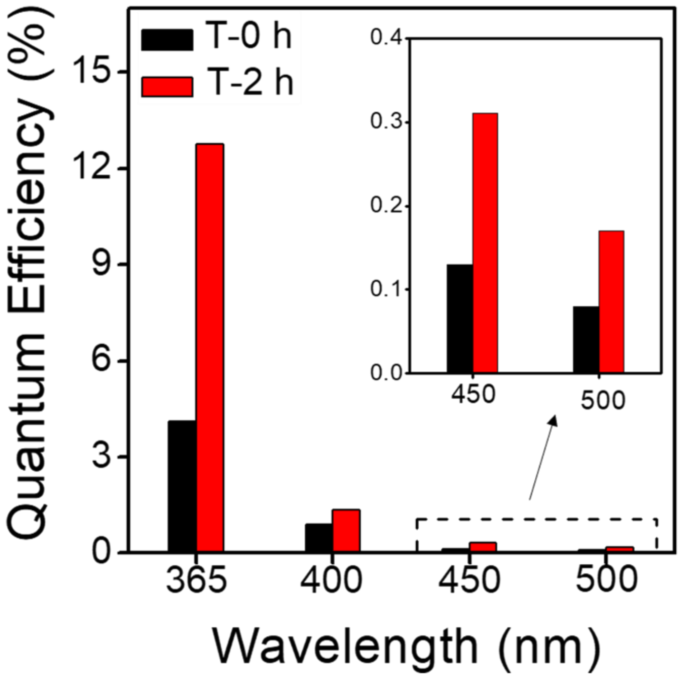

Figure S21. The AQE of T-x during the photocatalytic degradation of acetaldehyde under monochromatic light.

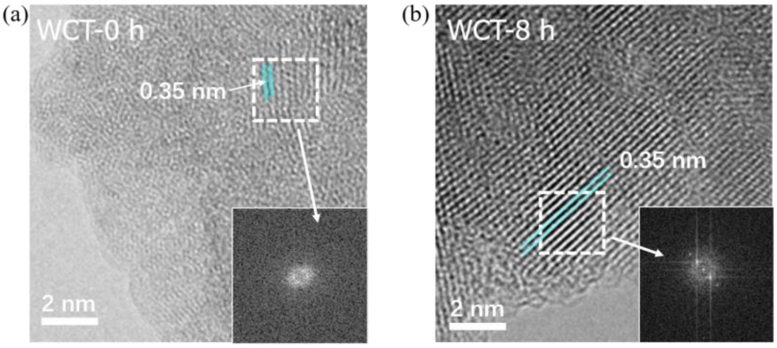

Figure S22. HRTEM images of (a) weak crystallized TiO_2(AB) and (b) weak crystallized TiO_2(AB) with SPP for 8 h. The insert photo is FFT images of the selected area in (a) and (b) marked with a white dot box.

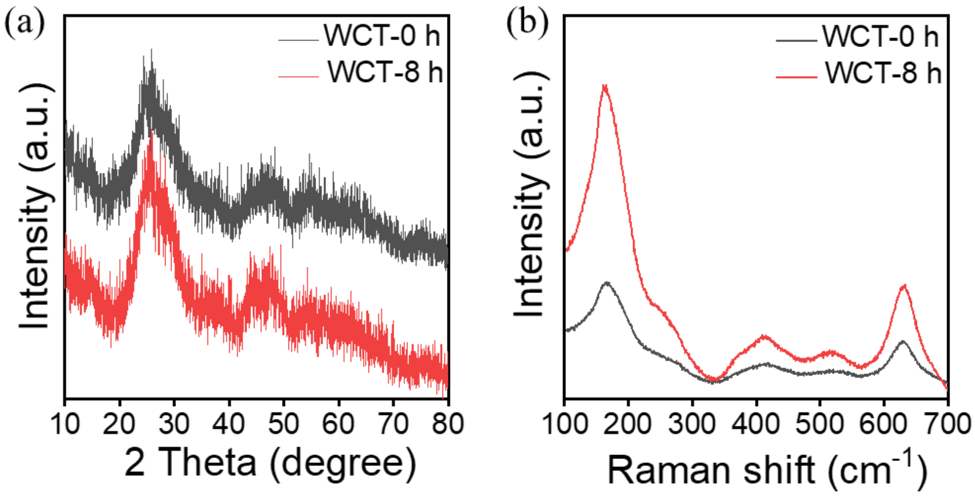

Figure S23. (a) XRD images and (b) Raman spectra of weak crystallized TiO_2(AB) and weak crystallized TiO_2(AB) with SPP for 8 h.

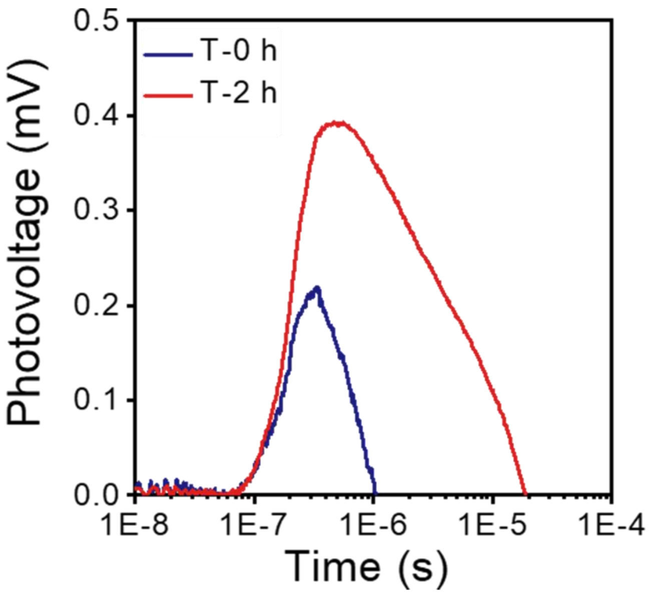

Figure S24. Transient photovoltage spectroscopy of T-x.

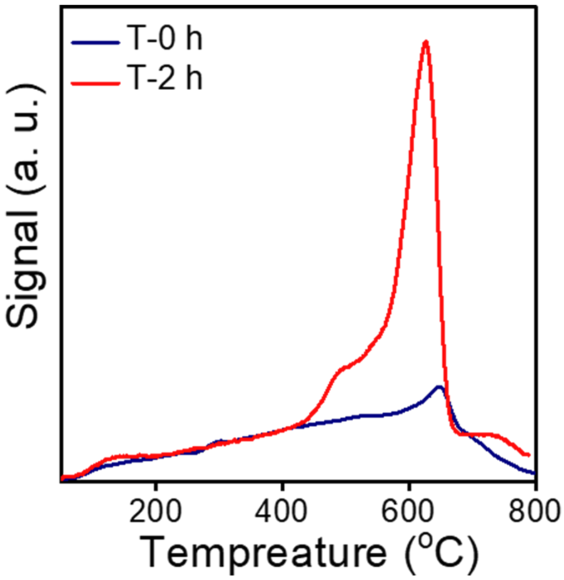

Figure S25. O_2-TPD profiles of T-x

4.2 The impact of processing time for TiO_2(AB) treated by SPP

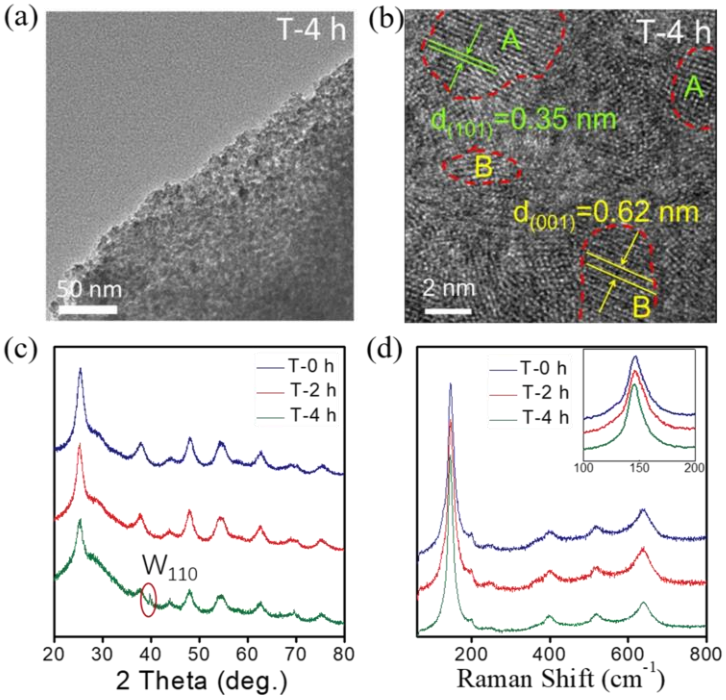

Figure S26. (a) TEM and (b) HRTEM images of T-4 h; (c) XRD patterns of T-x; (d) Raman spectra of T-x, the insert in (d) is the regional enlarged spectra of T-x.

The result of the TEM, the HRTEM, the XRD and the Raman suggest that the phase composition of TiO_2(AB) is not changed through the SPP method with the increasing of the time. The Raman spectral peaks of T-x have a trace shifting with the increased time of SPP, we supposed that the lattice disorder of TiO_2(AB) may be decreased.

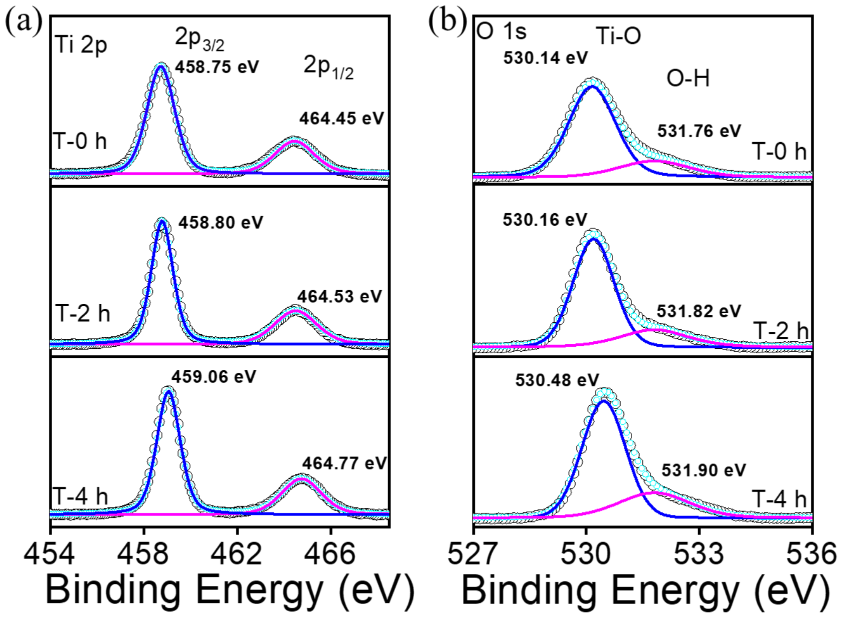

Figure S27. XPS spectra of (a) Ti 2p and (b) O 1s of T-x.

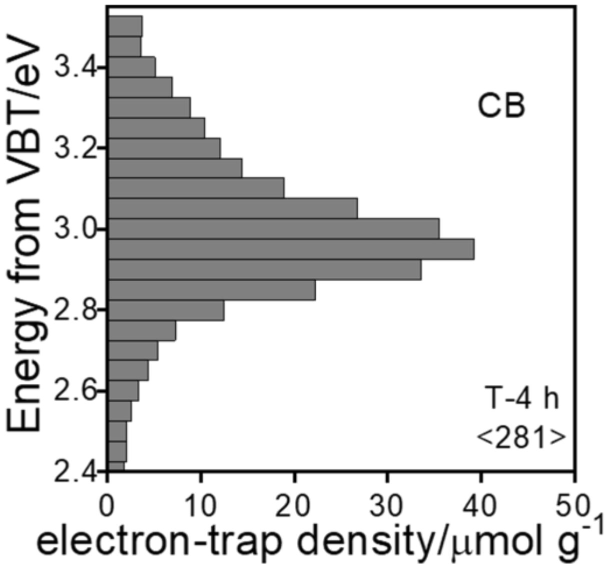

Figure S28. Reversed double-beam photoacoustic spectroscopy of T-4 h, Figure in <> denotes the total density of electron-trap with the unit of μmol g^{-1}.

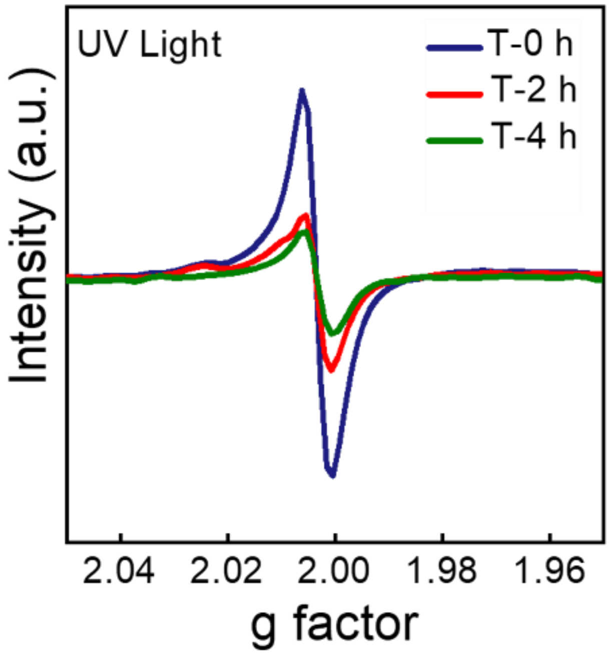

Figure S29. ESR spectra of T-x under 90 K.

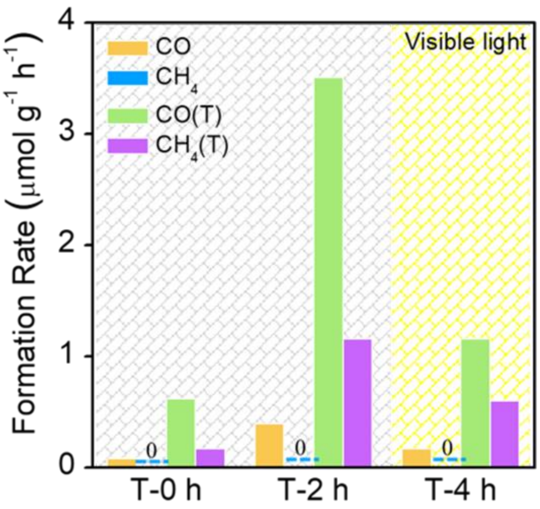

Figure S30. Photo- and photothermal catalytic activities (PC and PTC, respectively) of CO_2 reduction over T-x samples under visible light (λ≥420 nm).

For visible light photocatalysis, the formation rates of products are enhanced and follow an order: T-2 h>T-4 h>T-0 h. These results indicate SPP can activate TiO_2(AB) and SPP for 2 hours gives the best performance of CO_2 reduction. For visible light photothermal catalysis, all samples show significant enhancement of catalytic activity. As shown in Figure S30, the activity again follows the same order to that of photocatalysis: T-2 h>T-4 h>T-0 h.

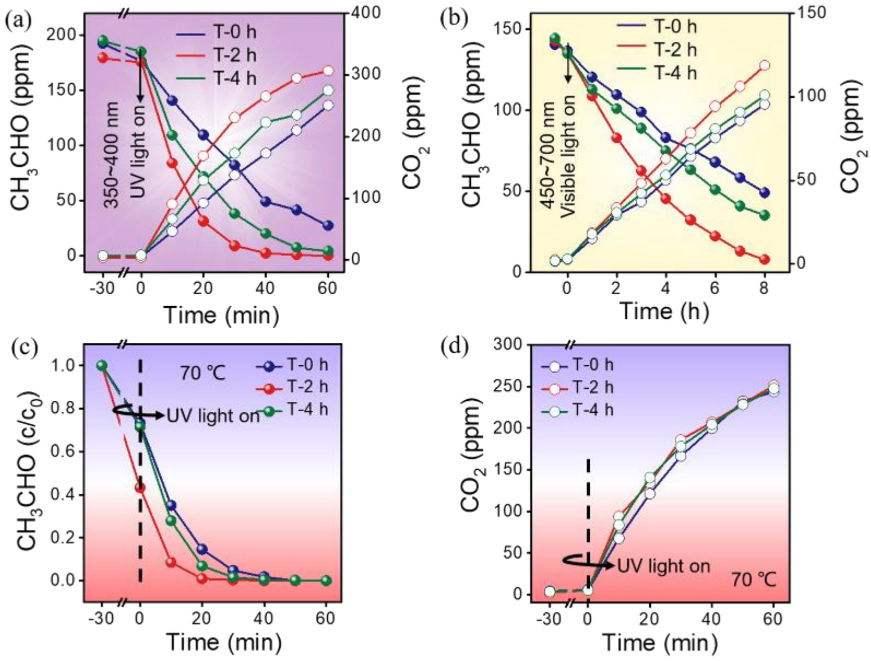

Figure S31. Time curves of acetaldehyde degradation and CO_2 generation over T-x under (a) UV light irradiation and (b) visible light irradiation (λ≥450 nm); (c) Time curves of acetaldehyde degradation and (d) CO_2 generation over T-x under UV light irradiation with heating of 70 ℃.

For photocatalysis and photothermal catalysis, TiO_2(AB) with increasing time of SPP treatment shows a volcano removal of acetaldehyde and evolution of CO_2. As shown in Figure S31, T-2 h shows the best photoactivity, while the photoactivity of T-4 h decreases.

4.3 The Effects of SPP for other TiO_2

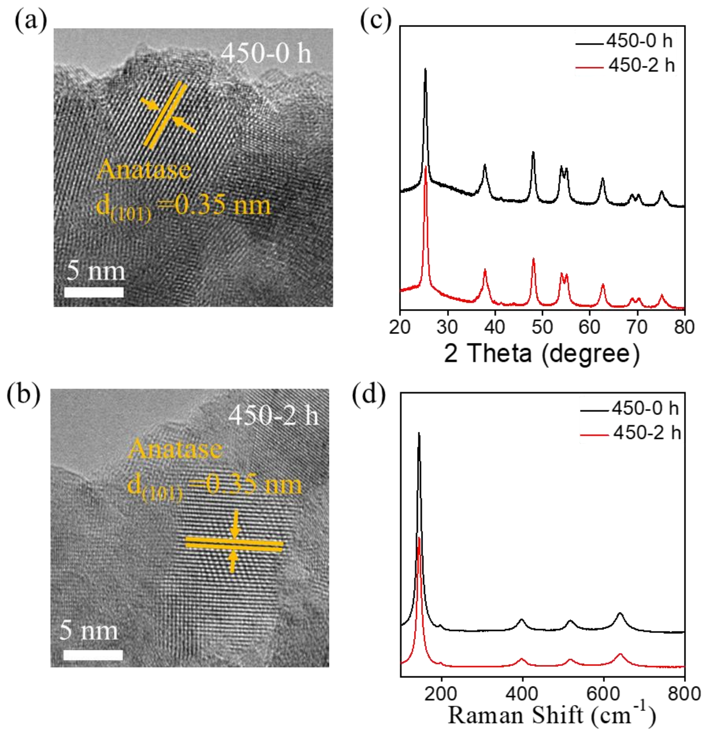

Figure S32. HRTEM images of (a) 450-0 h and (b) 450-2 h; (c) XRD patterns and (d) Raman spectra of 450-0 h and 450-2 h.

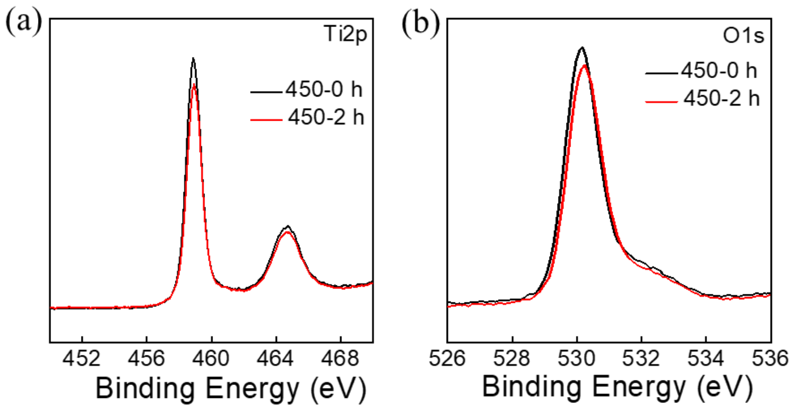

Figure S33. XPS spectra of (a) Ti 2p and (b) O 1s spectra of 450-0 h and 450-2 h.

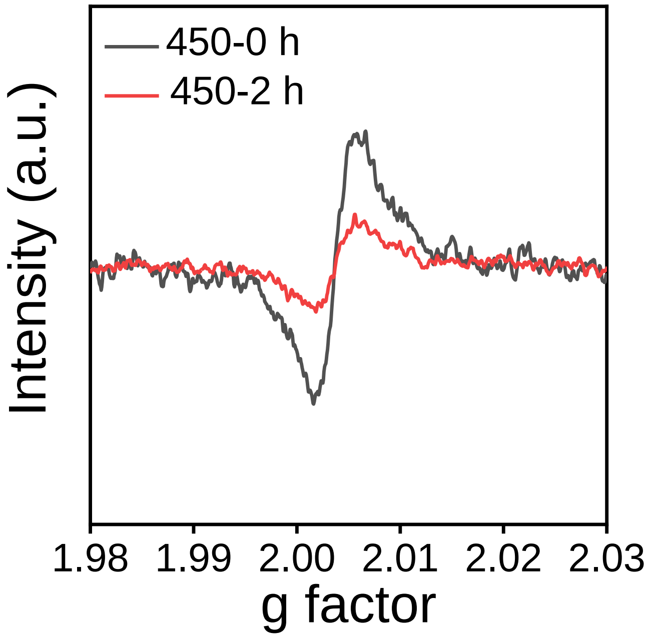

Figure S34. ESR spectra of 450-0 h and 450-2 h.

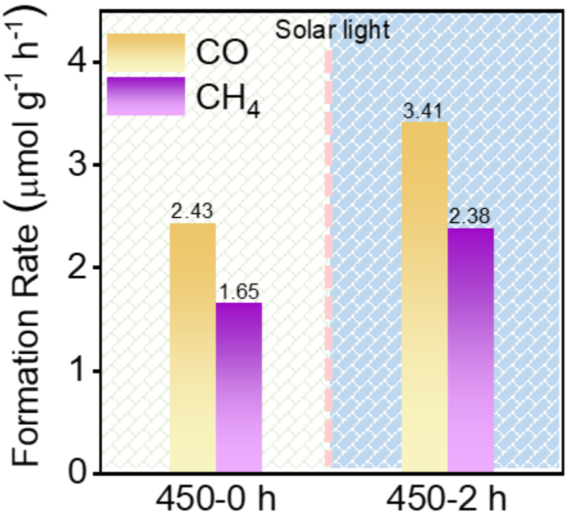

Figure S35. Photothermal catalytic activities (PTC) of CO_2 reduction over 450-0 h and 450-2 h samples under solar light.

4.4 The Effects of SPP for other semiconductor oxides

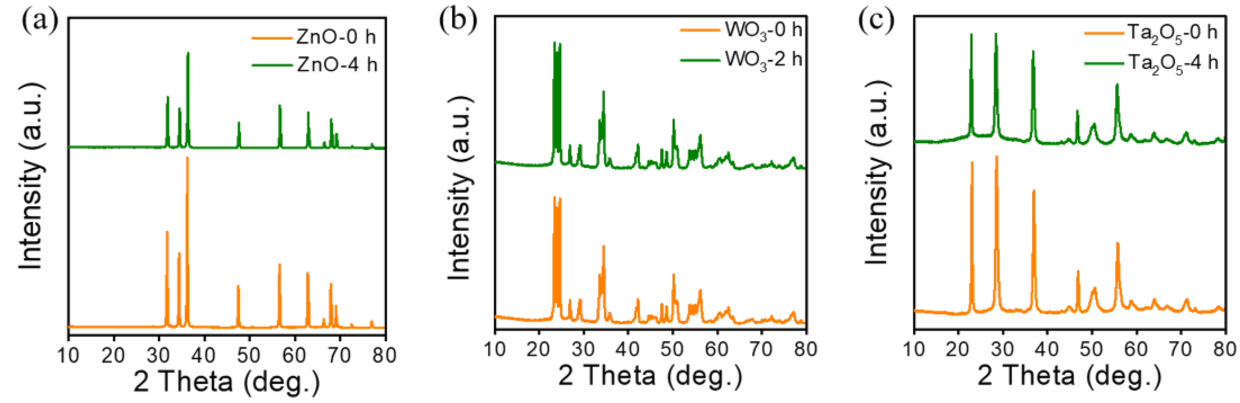

Figure S36. XRD patterns of different semiconductor oxide samples untreated or treated by SPP: (a) ZnO-0 h and ZnO-4 h; (b) WO_3-0 h and WO_3-2 h; (c) Ta_2O_5-0 h and Ta_2O_5-4 h.

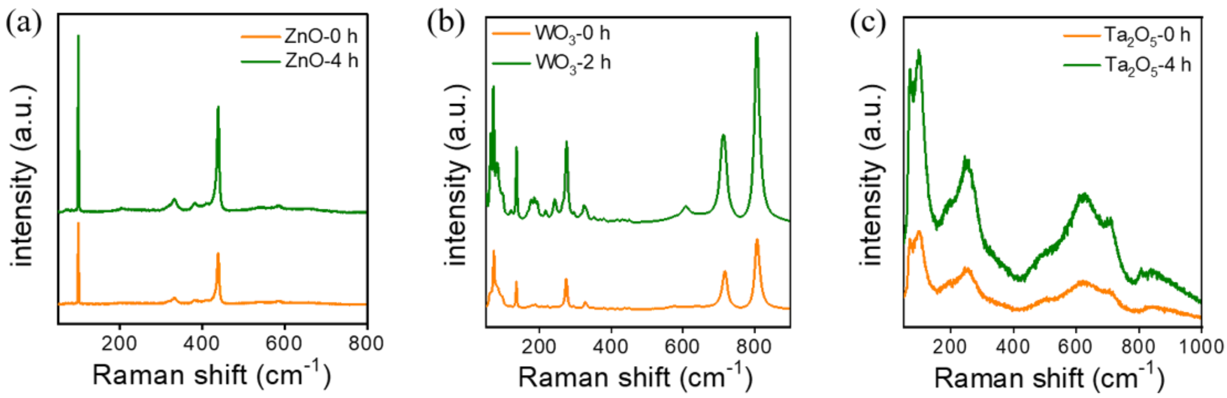

Figure S37. Raman spectra of different semiconductor oxide samples untreated or treated by SPP: (a) ZnO-0 h and ZnO-4 h; (b) WO_3-0 h and WO_3-2 h; (c) Ta_2O_5-0 h and Ta_2O_5-4 h.

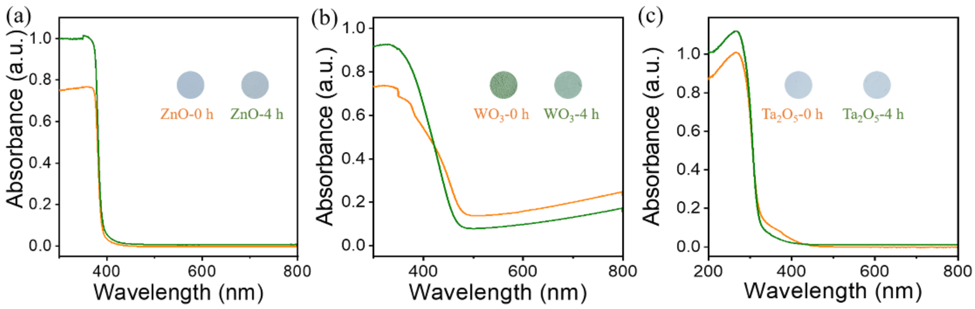

Figure S38. The ultraviolet-visible absorption spectra of different semiconductor oxide samples untreated or treated by SPP: (a) ZnO-0 h and ZnO-4 h; (b) WO_3-0 h and WO_3-2 h; (c) Ta_2O_5-0 h and Ta_2O_5-4 h; the inserts are their photo.

To determine whether SPP is applicable to other semiconductor oxides, we have selected three commercial semiconductor oxides as following: ZnO, WO_3 and Ta_2O_5. The characterization of XRD and Raman prove that the phase composition of them is not changed after SPP treatment. Figure S38 displays the diffuse reflectance UV−vis spectrum of untreated and treated semiconductor oxides. Before and after the SPP treatment, the intrinsic band edge of ZnO and Ta_2O_5 has no noticeable shift. WO_3 treated by SPP shows same results with TiO_{2-x}.

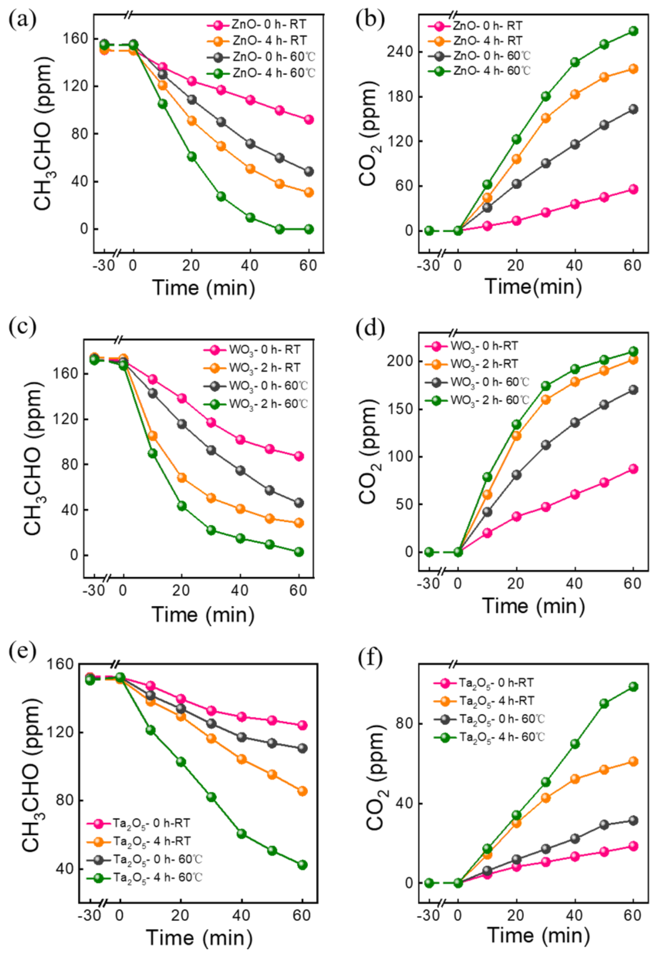

Figure S39. Time curves of acetaldehyde degradation and CO_2 generation over different semiconductor oxide samples untreated or treated by SPP: (a)-(b) ZnO-0 h and ZnO-4 h; (c)-(d) WO_3-0 h and WO_3-2 h; (e)-(f) Ta_2O_5-0 h and Ta_2O_5-4 h under UV light irradiation at room temperature (RT) and 70 ℃.

The photocatalytic activity of untreated and treated semiconductor oxides was assessed by monitoring the degradation of acetaldehyde gas under UV light irradiation, and the course of degradation of acetaldehyde and the evolution of CO_2 were displayed in Figure S39. The activity follows the order to that of photocatalysis: treated semiconductor oxides > untreated semiconductor oxides. The activity of photothermalcatalysis show the same trend with that of photocatalysis. Therefore, SPP is also generally applicable to other semiconductor oxides.

Table S1. Comparison of photocatalytic activity of CO_2 reduction over TiO_2-based catalysts.

Catalyst	Reaction conditions	Products	Max CH_4 Yield μmol g^{-1} h^{-1}	Max CO Yield μmol g^{-1} h^{-1}	Selectivity of CH_4	Ref.
TiO_2-O_V<br>TiO_2-O_V-Co	50 mg cat., 1.33 bar CO_2, 393 K, 2 mL H_2O, UV light.	CH_4, CO	0.4829<br>10.051	13.362<br>16.403	12.63%<br>71.02%	[11]
H-TiO_2-O_V	50 mg cat., 2 bar CO_2, 6 mL H_2O, solar light.	CH_4, CO, H_2	16.2	4.2	79%	[12]
H-TiO_2-O_V	50 mg cat., 2 bar CO_2, 6 mL H_2O, solar light.	CH_4, CO, H_2	14.3	4.8	74%	[13]
P25	20 mg cat., 0.2 Mpa CO_2, 4.0 mL H_2O, UV-Vis light.	CH_4, CO, H_2	1.2	2.5	65.7%	[14]
TiO_2	100 mg cat., 300 W Xe lamp, atmospheric pressure		1.35			[15]
Reduced {001}-TiO_{2-x}	30 mg cat., 300 W Xe lamp, AM1.5	CH_4, CO,	< 0.3	<0.18		[16]
Ti^{3+}-brookite TiO_2	300 W Xe lamp, Vis-light, continuous CO_2	CH_4, CO,	11.9	23.5	66.95%	[17]
Ti^{3+}-rutile TiO_2	100 mg cat., 300 W Xe lamp, Vis-light, 1 atm	CH_4, CO,	< 0.7	< 0.1		[18]
P25 particles	1000 W Xe lamp, λ < 700 nm, 343 K	CH_4, CO, H_2	0.1			[19]
TiO_2	200 mg cat., 0.2 MPa CO_2, 323 K, 4 mL H_2O, UV−vis light	CH_4, CO, H_2	0.38	1.2	55.88%	[20]
TiO_2 microsphere	200 mg cat., CO_2, 100 μL H_2O, Hg UV lamp	CH_4, CO, H_2	0.94	2.32	19.68%	[21]
Ti^{3+}-anatase TiO_2<br>Ti^{3+}-rutile TiO_2	CO_2, 1 mL H_2O,<br>A 300 W Xe lamp	CH_4, CO	5.57<br>1.63	5.08<br>1.06	81.43%<br>86.01%	[22]
T-2 h	50 mg cat., 1.33 bar CO_2, 393 K, 2 mL H_2O, UV light.	CH_4, CO,	38.99	11.93	55.0%	This work

Table S2. Compared the relationship between products and reaction conditions in photocatalytic CO_2 reduction

Photocatalyst	Light source	Experimental condition	Main products	Reference
Single-crystalline Zn_2GeO_4 nanobelts	300 W Xe lamp	CO_2 and H_2O vapor	CH_4	[23]
Defective TiO_2 (anatase, rutile, and brookite)	150 W solar simulator	CO_2 and H_2O vapor	CH_4	[24]
Codoped TiO_2	300 W Xe lamp ($\lambda > 420$ nm)	CO_2 and H_2O vapor	CO, CH_4	[25]
Anatase TiO_2	300 W Xe lamp	CO_2 and H_2O vapor	CH_4	[26]
Cu/Degussa P25	150W solar simulator	CO_2 and H_2O vapor	CO, CH_4	[27]
WO_3	300 W Xe lamp	CO_2 and H_2O vapor	CH_4	[28]
Titanate nanosheet assembled Yolk@Shell Microspheres	150 W Xe lamp ($\lambda > 420$ nm)	CO_2 saturated water ($NaHCO_3$+HCl)	CH_3OH	[29]
$AgBr/TiO_2$	150 W Xe lamp ($\lambda > 420$ nm)	CO_2 saturated KOH solution	CH_4, CH_3OH, C_2H_5OH, CO	[30]
Defective single-unit-cell $BiVO_4$ layers	300 W Xe lamp (AM1.5)	CO_2 saturated water	CH_3OH	[31]
Ag /TiO_2	8 W Hg lamp	CO_2 bubbled solution	CH_4, CH_3OH	[32]
Ag /TiO_2	500 W Xe lamp ($\lambda > 420$ nm)	CO_2 saturated H_2O	CH_3OH	[33]

Additionally, the products of C1 are dependent on the experimental conditions. In general, the reported researches on CO_2 reduction with H_2O can be classified into two types (summarized in Table S2): CO_2 reduction by H_2O vapor and CO_2 reduction by H_2O liquid. Therein, CO_2 reduction by H_2O vapor mainly produces CO and CH_4. Differently, CO_2 reduction by H_2O liquid mainly produces CH_3OH. In our work, the photothermal catalytic reduction of CO_2 is performed in H_2O vapor. CO and CH_4 are main products, which is in consistence with previously reported works.

5. References:

[1] A. Nitta, M. Takashima, M. Takase, B. Ohtani, *Catal. Today* 2019*, 321*, 2.

[2] A. Nitta, M. Takashima, N. Murakami, M. Takase, B. Ohtani, *Electrochimica Acta*, 2018, *264*, 83.

[3] A. Nitta, M. Takase, M. Takashima, N. Murakami, B. Ohtani, *Chem. Comm.* 2016, *52*, 12096.

[3] G. Kresse, J. Furthmüller, *Comp. Mater. Sci.* 1996, 6, (1), 15.

[4] P. E. Blöchl, *Phys. Rev. B* 1994, *50*, 17953.

[5] J. P. Perdew, J. A. Chevary, S. H. Vosko, K. A. Jackson, M. R. Pederson, D. J. Singh, C. Fiolhais, *Phys. Rev. B* 1992, 46, 6671.

[6] H. J. Monkhorst, J. D. Pack, *Phys. Rev. B* 1976, 13, 5188.

[7] E. Skulason, T. Bligaard, S. Gudmundsdottir, F. Studt, J. Rossmeisl, F. Abild-Pedersen, T. Vegge, H. Jonsson, J. K. Norskov, *Phys. Chem. Chem. Phys.* 2012, 14, 1235.

[8] A. A. Peterson, F. Abild-Pedersen, F. Studt, J. Rossmeisl, J. K. Nørskov, *Energy Environ. Sci.* 2010, *3*, 1311.

[9] Q. Tang, Y. Lee, D. Y. Li, W. Choi, C. W. Liu, D. Lee, D. E. Jiang, *J. Am. Chem. Soc.* 2017, *139*, 9728.

[10] L. Liu, Y. Y. Peter, X. Chen, S. S. Mao, D. Z. *Shen*, *Phys. Rev. Lett.* 2013, *111*, 065505.

[11] Y. Li, C. Wang, M. Song, D. Li, X. Zhang, Y. Liu, *Appl. Catal. B Environ.* 2019, *243*, 760.

[12] G. Yin, X. Huang, T. Chen, W. Zhao, Q. Bi, J. Xu, Y. Han, F. Huang, *ACS Catal.* 2018, *8*, 1009.

[13] G. Yin, Q. Bi, W. Zhao, J. Xu, T. Lin, F. Huang, *ChemCatChem* 2017, *9*, 4389.

[14] Q. Zhai, S. Xie, W. Fan, Q. Zhang, Y. Wang, W. Deng, Y. Wang, *Angew. Chem. Int. Ed.* 2013, *52*, 5776.

[15] J. Yu, J. Low, W. Xiao, P. Zhou, M. Jaroniec, *J. Am. Chem. Soc.* 2014, *136*, 8839.

[16] W. Fang, L. Khrouz, Y. Zhou, B. Shen, C. Dong, M. Xing, S. Mishra, S. Daniele, J. Zhang, *J. Phys. Chem. Chem. Phys.* 2017, *19*, 13875.

[17] X. Xin, T. Xu, L. Wang, C. Wang, *Sci. Rep.* 2016, *6*, 23684.

[18] K. Sasan, F. Zuo, Y. Wang, P. Feng, *Nanoscale* 2015, *7*, 13369.

[19] F. Saladin, L. Forss, I. Kamber, *J. Chem. Soc. Chem. Commun.* 1995, *5*, 533.

[20] S. Xie, Y. Wang, Q. Zhang, W. Deng, Y. Wang, *ACS Catal.* 2014, *4*, 3644.

[21] B. Fang, A. Bonakdarpour, K. Reilly, Y. Xing, F. Taghipour, D. P. Wilkinson, *ACS Appl. Mater. Interfaces* 2014, *6*, 15488.

[22] M. Xing, Y. Zhou, C. Dong, L. Cai, L. Zeng, B. Shen, L. Pan, C. Dong, Y. Chai, J. Zhang, Y. Yin, *Nano Lett.* 2018, *18*, 3384.

[23] Q. Liu, Y. Zhou, J. Kou, X. Chen, Z. Tian, J. Gao, S. Yan, Z. Zou, *J. Am. Chem. Soc* 2010, *132*, 14385.

[24] L. Liu, H. Zhao, J. M. Andino, Y. Li, *ACS Catal.* 2012, *2*, 1817.

[25] T. Wang, X. Meng, G. Liu, K. Chang, P. Li, Q. Kang, L. Liu, M. Li, S. Ouyang, J. Ye, *J. Mater. Chem. A* 2015, *3*, 9491.

[26] J. Yu, J. Low, W. Xiao, P. Zhou, M. Jaroniec, *J. Am. Chem. Soc.* 2014, *136*, 8839.

[27] L. Liu, F. Gao, H. Zhao, Y. Li, *Appl. Catal., B* 2013, *134*, 349.

[28] X. Chen, Y. Zhou, Q. Liu, Z. Li, J. Liu, Z. Zou, *ACS Appl. Mater. Interfaces* 2012, *4*, 3372.

[29] S. Liu, J. Xia, J. Yu, *ACS Appl. Mater. Interfaces* 2015, *7*, 8166.

[30] M. A. Asi, C. He, M. Su, D. Xia, L. Lin, H. Deng, Y. Xiong, R. Qiu, X. Z. Li, *Catal. Today* 2011, *175*, 256.

[31] S. Gao, B. Gu, X. Jiao, Y. Sun, X. Zu, F. Yang, W. Zhu, C. Wang, Z. Feng, B. Ye, Y.

Xie, *J. Am. Chem. Soc.* 2017, *139*, 3438.

[32] K. Koci, K. Mateju, L. Obalova, S. Krejcikova, Z. Lacny, D. Placha, L. Capek, A. Hospodkova, O. Solcova, *Appl. Catal., B* 2010, *96*, 239.

[33] E. Liu, L. Kang, F. Wu, T. Sun, X. Hu, Y. Yang, H. Liu, J. Fan, *Plasmonics* 2014, *9*, 61.
